# Exploring the impact of plant growth-promoting bacteria in alleviating stress on *Aptenia cordifolia* subjected to irrigation with recycled water in multifunctional external green walls

**DOI:** 10.1186/s12870-024-05511-9

**Published:** 2024-08-24

**Authors:** Mansoure Jozay, Hossein Zarei, Sarah Khorasaninejad, Taghi Miri

**Affiliations:** 1 Horticultural Sciences Department, Faculty of Plant Production, Gorgan University of Agricultural Sciences and Natural Resources, Gorgan, Iran; 2 School of Chemical Engineering, University of Birmingham, Birmingham, UK

**Keywords:** Antioxidant activity, External green wall, Gray water, Ornamental plant, Oxidative stress, Wastewater

## Abstract

**Background:**

Rapid urbanization and population growth exert a substantial impact on the accessibility of drinking water resources, underscoring the imperative for wastewater treatment and the reuse of non-potable water in agriculture. In this context, green walls emerge as a potential solution to augment the purification of unconventional waters, simultaneously contributing to the aesthetic appeal and enjoyment of urban areas. This study aims to optimize water management in green walls by investigating the impact of bacterial strains on the biochemical properties and performance of the ornamental accumulator plant, *Aptenia cordifolia*, grown with various unconventional water sources. The experiments were designed as split plots based on a completely randomized block design with three replications. The main factor was recycled water with three levels (gray water, wastewater from the Kashfroud region of Mashhad, and urban water (control)). The sub-factor included different bacterial strains at four levels, composed of various bacteria combinations, (B1: *Psedoumonas flucrecens* + *Azosporillum liposferum* + *Thiobacillus thioparus* + *Aztobactor chorococcum*, B2: *Paenibacillus polymyxa* + *Pseudomonas fildensis* + *Bacillus subtilis* + *Achromobacter xylosoxidans* + *Bacillus licheniform*, B3: *Pseudomonas putida* + *Acidithiobacillus ferrooxidans* + *Bacillus velezensis* + *Bacillus subtilis* + *Bacillus methylotrophicus* + *Mcrobacterium testaceum*, and the control level without bacterial application (B0).

**Result:**

The findings revealed significant differences at the 5% probability level across all morphophysiological traits, including plant height, the number and length of lateral branches, growth index, and plant coverage. Moreover, superior morphophysiological traits were observed in plants cultivated in substrates inoculated with wastewater irrigation. Substrates inoculated with bacteria exhibited the highest relative water content (RWC) and chlorophyll levels, coupled with the lowest relative saturation deficit (RSD), electrolyte leakage (EL), and carotenoid levels. Furthermore, plant growth-promoting bacteria (PGPB), from a biochemical perspective, were associated with increased carbohydrates, total protein, and anthocyanin. They also contributed to controlling oxidative stress caused by free radicals by enhancing the activity of antioxidant enzymes, such as guaiacol peroxidase (GPX), polyphenol oxidase (PPO), ascorbate peroxidase (APX), and peroxidase (POD), while reducing catalase enzyme (CAT) activity. This led to increased resistance to stress, as evidenced by a decrease in malondialdehyde and proline levels. The study concludes that the MIX B3, being both ecofriendly and economical, represents an effective strategy for mitigating the adverse effects of wastewater on plants.

**Conclusion:**

This study showed that plant irrigation using wastewater increases the levels of proline, phenols and oxidative stress. However, the application of plant growth promoting bacteria (PGPB) reduced oxidative damage by increasing antioxidant activity and decreasing proline and phenol levels. These findings show the potential of bacterial treatments to improve plant growth and reduce adverse effects of recycled water irrigation.

## Background

Urban environments, with their current unsustainable developments, pose significant challenges that require fundamental environmental reforms. The primary reasons for these challenges include the purification of pollutants, urban heat islands, the expansion of impervious surfaces, climate change, loss of biodiversity, and the aesthetic damage associated with current urban transformations [1]. However, natural and non-built urban environments have the capacity for ecological restoration and improvement. Unfortunately, the unsustainable approaches of urban development have deprived us of these options [2]. To address this dilemma, Artmann and Sartison [3] proposed a nature-based solution. One of these solutions is sustainable green infrastructure in cities, including exterior green walls.

Considering that horizontal expansion of green spaces may not be feasible due to limited vacant land, vertical development of gardens remains the only viable solution in theory to fulfill this objective. The severe shortage of space in today's densely populated urban environments underscores the need for future cities to explore the expansion of rooftops and green walls [4]. Vertical green walls can be considered as sustainable environmental technologies that offer numerous economic, environmental, and social benefits and are also regarded as urban lungs [5]. However, the fundamental potential of these infrastructures, in terms of sustainable building elements, has received less attention in research with the aim of enhancing their environmental efficiency and multifunctionality [6].

The high water demand of green systems is a limiting factor for their development in water-scarce regions. To minimize water requirements, it is advisable to opt for drought-tolerant and heat-resistant plant species [7]. For example, *Aptenia cordifolia* is a CAM (Crassulacean Acid Metabolism) plant that is drought-resistant and also capable of phytoremediation. *Aptenia cordifolia* belonging to the Aizoaceae family, is a succulent and perennial species widely employed in green spaces in southern Iran. It is known as a hardy, flowering, perennial, trailing plant. In Iran it is also introduced as Ice Flower. *A. cordifolia* is resistant to heat and drought. It has light pink, white and red flowers that bloom throughout the year. The solitary to clustered flowers arise in leaf axils and generally open during the day. This plant needs full sun exposure.

Another solution can be the utilization of alternative water sources such as graywater and wastewater [5]. Since green walls are considered an environmental innovation, it is necessary to incorporate the possibility of rainwater harvesting and the reuse of recycled water [8].

Soil pollution with heavy metals, originating from fertilizers and non-conventional water sources used in urban green spaces, presents a significant issue due to the potential transfer of these contaminants to agricultural products in cities. Therefore, multiple challenges hinder the sustainability of this concept in urban environments, requiring effective planning to address them. Important factors such as agricultural and horticultural practices, including growth-promoting bacteria, design elements, and plant characteristics, play a crucial role in soil filtration [9] and the removal of pollutants. Recently, it has been reported that planting crops in Substrate containing certain growth-promoting bacteria can efficiently fulfill their nutrient requirements and reduce the need for chemical fertilizers [10]. Research on biofertilizers includes various types of microorganisms that can convert less accessible forms of essential nutrients into accessible forms through biological processes. This leads to the development of better root systems and seed germination [11]. Liu et al., [12] isolated forty-one bacterial strains from the rhizosphere soil and root tissues of five plant species (*Artemisia argyi* Levl.، *Gladiolus gandavensis* Vaniot Houtt، *Boehmeria nivea* L.، *Veronica didyma* Tenore و*Miscanthus floridulonizing* Lab). Their results showed that among the bacteria, two strains, *Klebsiella michiganensis* TS8 and *Lelliottia jeotgali* MR2, exhibited higher tolerance to cadmium and were highly successful in cadmium phytoremediation of the soil. TS8 increased plant height and the dry weight of leaves from 9.39 to 1.99. It appears that strains of Pseudomonas, Mycobacterium, Staphylococcus, Micrococcus, Bacillus, Paenibacillus, and Klebsiella were widely used solely for phytoremediation, and the combined application of these strains were more successful than their individual application [13]. Some native plants and grasses also have the potential for phytoremediation in metal-contaminated water. Phytoremediation through biostimulation is a promising approach that can enhance the synergistic effects of microorganisms and plants [14].

On one hand, the most significant challenge is population growth and the increasing water demand for economic activities, particularly agriculture. Water scarcity is a major concern in densely populated urban areas. On the other hand, the disposal of graywater and urban wastewater can contaminate surface and groundwater sources. In this regard, green walls can improve the treatment of non-conventional waters, enhance the hydrological cycle, and increase the beauty and enjoyment of urban areas [15]. The benefits of using ornamental plant coverings with distinct aesthetic features are evident in satisfying the visual preferences of the community and providing pleasant landscape value [16]. Using alternative ornamental species in constructed wetlands (CWs), represents an effective system for pollutant removal [17]. Additionally, the use of these systems can significantly improve the visual quality of the landscape, which is often undervalued but has positive social and psychological impacts on people's daily lives [18].

One of the important aspects of non-conventional water is graywater and wastewater. Graywater refers to wastewater generated from laundry, toilets, showers, baths (also known as light graywater), and, in some cases, kitchen sinks and dishwashers (known as dark graywater). Light graywater is produced in significant amounts (45% to 60% of domestic wastewater) and contains a lower pollutant load compared to mixed domestic wastewater [19]. For this reason, considerable efforts have been made to reuse it on-site. Additionally, treated wastewater has good nutrient value that can enhance plant growth, reduce fertilizer consumption, and increase the productivity of nutrient-depleted soils [20, 21].

Given the challenges posed by population growth and increased water demand for economic activities, particularly in agriculture, urban areas face significant hurdles. Freshwater scarcity emerges as a primary concern in these regions, where the disposal of graywater and urban wastewater poses a potential threat to surface and groundwater sources, exacerbating the issue. While green walls can effectively treat polluted water, enhance the hydrological cycle, and beautify urban areas, they may also introduce stress conditions. These stressors arise from microclimate factors and the use of unconventional water for plant growth, potentially increasing the transfer of heavy metals and other non-biological stresses Additionally, the use of wastewater in urban agriculture requires additional caution. Therefore, the primary objective of this study is to investigate the feasibility of utilizing organic biological fertilizers to improve soil organic matter content, providing a sustainable alternative to chemical fertilizers. The goal is to mitigate existing stresses and promote environmentally friendly soil management practices.

## Methods

### The study area and site

**Fig. 1:**
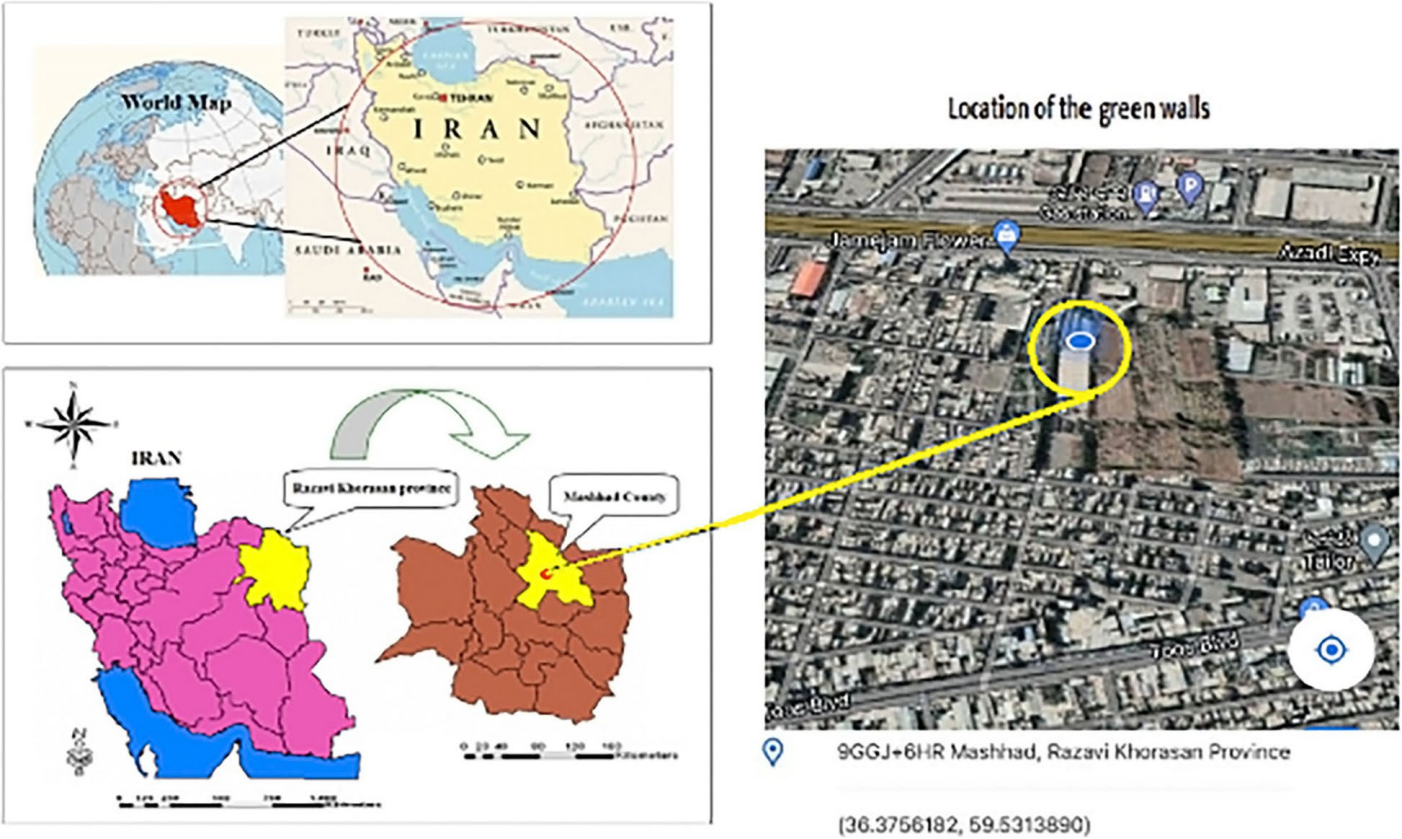
Location of the study site in Mashhad

This research was conducted in Mashhad, located in northeastern Iran. Mashhad is the capital of Khorasan Razavi province and the second largest and most populous city in Iran. It has a semi-arid climate with cold winters and hot, dry summers (elevation of 995 m above sea level, geographical coordinates 36 degrees 18 min north, 59 degrees 36 min east). The average annual precipitation is approximately 255 mm. The average minimum and maximum temperatures annually are -4 and 22 degrees Celsius, respectively, and the relative humidity is reported to be about 40% [22]. The precise location of the experiment was on a 15 m wall in the outdoor area of the Armgan Greenhouse, located in the northern part of Mashhad (Fig. 1).

### The green wall systems

To conduct this research, vertical cultivation panels (mesh made of 5 mm steel wire) were installed at a suitable distance from shading factors, facing east–west. The vertical cultivation system used in this project was called the "Almich" system (initially developed in Malaysia). The experimental units consisted of Almich green wall pots, plastic pots made of new or recycled polypropylene, with dimensions of approximately 20 × 20 and a depth of about 21 cm. Leca was used as a drainage layer at the bottom of the pots, followed by a layer of geotextile as a soil filter.

**Fig. 2:**
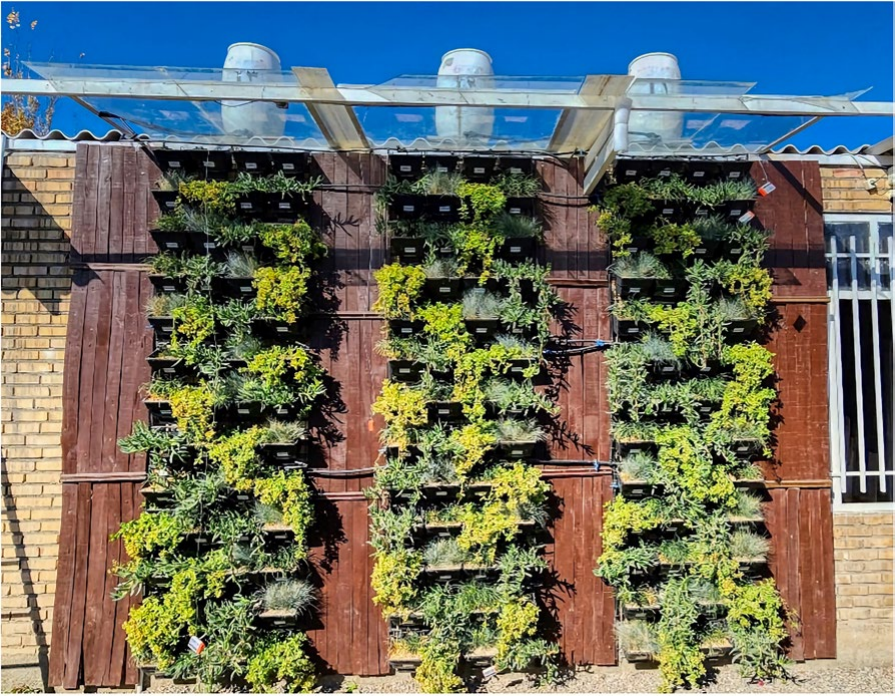
A view of the experiment’s external green walls

In each plot, two plants of each tested species were cultivated. This study was conducted on an exterior green wall from March to December 2022. Each wall consisted of two panels measuring 44 × 106 cm, and each set of three walls constituted one replication of the experiment. Each wall had 4 vertical rows and 4 horizontal rows, resulting in a total of 16 plots (experimental units) per wall. Considering A. cordifolia and the mentioned treatments with three replications, the first experiment included 48 experimental units (Fig. 2).

#### The experiment

This experiment aimed to investigate the impact of irrigation water quality and different bacterial strains on the growth and performance of selected ornamental plants with phytoremediation characteristics, while also measuring the water consumption of these species under green wall conditions. This research was implemented as split-plot layout, based on a randomized complete block design with three replications from March to December 2022. The organic matter content in this experiment was 20%, which is very close to the maximum recommended 30% organic matter content (OC) by FLL (2018) [23].

The growth medium used in all experimental units was the same and consisted of the following components: 25% cocopeat [24], 5% vermicompost [25], 55% perlite [26], 10% vermiculite [27], and 5% zeolite [28].

##### Unconventional water treatments

The main-factor included graywater collected from rainwater, a twin sink designed for fruit and vegetable washing, wastewater from the Kashafrood region and urban water (control). The main factor was applied in three main tank reservoirs and irrigated to the plants in the form of drip irrigation, with irrigation levels at around 80% of the field capacity, adjusted based on the flow rate of the drip emitters.

##### Treatment of plant growth-promoting *bacteria*

In this research, growth-promoting bacteria were utilized. Apart from fulfilling the plant's requirements for chemical fertilizers, these bacteria also absorbed heavy metals from the soil [29, 30]. Previous research suggests that the combined application of these bacterial strains has been more effective than their individual application [30]. The sub-factor of different biological strains of bacteria at four levels: Mix1 (*Psedoumonas flucrecens* + *Azosporillum Liposferum* + *Thiobacillus thioparus* + *Aztobactor chorococcum*), Mix2 (*Paenibacillus polymyxa* + *Pseudomonas fildensis* + *Bacillus subtilis* + *Achromobacter xylosoxidans* + *Bacillus licheniformis*), Mix3 (*Pseudomonas putida* + *Acidithiobacillus ferrooxidans* + *Bacillus velezensis* + *Bacillus subtilis* + *Bacillus methylotrophicus* + *Mcrobacterium testaceum*) and B0- control (without bacterial inoculation).

Each plant received 20 cc of the biofertilizer. The bacteria utilized in this experiment were procured from the Soil Biology and Biotechnology Laboratory at the Golestan Agricultural and Natural Resources Research Center LBSG, specifically identified by isolate number 041011 in the laboratory bank. The bacteria were extracted from the rhizosphere of agronomy plants like soybean and wheat using the method of Ju et al. [31]. The full scientific name and strains of the bacterial treatments in this study are given in Abbreviation. Except for the non-inoculated controls, the substrates around each plant specimen were watered and inoculated with 20 ml of bacterial suspension to obtain a bacterial concentration of 108 CFU/ml. The liquid was injected evenly around the plant root zone and the substrate surface around each plant using a syringe two weeks after planting. This method was selected to ensure an even distribution of the bacterial treatment in the substrate.

##### Selected accumulator ornamental plant

**Fig. 3:**
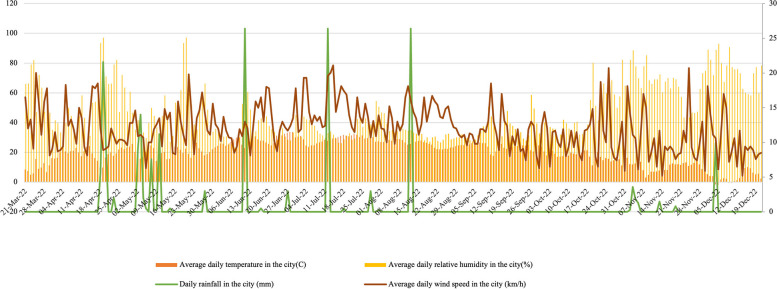
Trends in Temperature, Relative Humidity, Precipitation, and Wind Speed in the Experimental Months of the Year 2022 in Mashhad City

This plant, especially in phytoremediation, has effective applications for pollution extraction. They may act as a phytostabilizer, particularly in areas affected by metals [32] (Fig. 3). The selection of *A. Cordifolia* for the experiment was based on its ornamental qualities and accumulation characteristics. Additionally, this plant belongs to the CAM family, and in accordance with the traits of the Crassulaceae pathway, it exhibits rapid growth, high biomass production, robust root development, and high water use efficiency. These attributes enable effective pollutant removal, making it suitable for use in urban green walls. The *Aptenia cordifolia* seedlings utilized in this experiment were sourced from regions conducive to their growth within the country (Iran). Specifically, they were obtained from the Shandiz greenhouse in Mashhad, where such seedlings are abundantly produced.

**Fig. 4:**
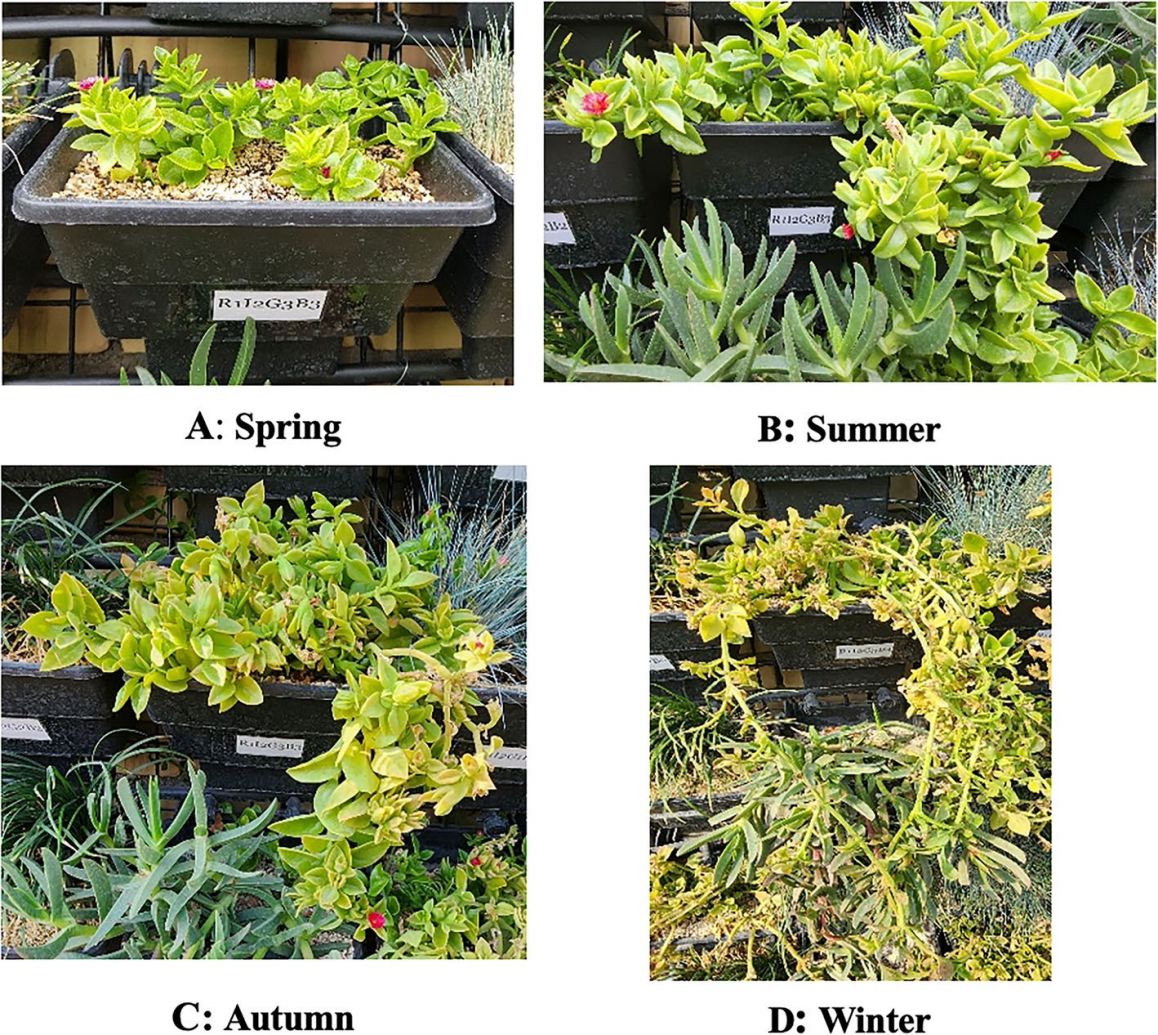
The appearance conditions of the four experimental plants studied in the under B3 and I2 treatments **A**, **B**, **C** and **D** during spring/summer/autumn and winter season!

The substrate was first passed through a 2 mm sieve after air drying. Physical and chemical characteristics were determined using standard laboratory methods as described in the subsequent sentences. Acidity and conductivity were measured using the extract and saturated mud [33], and the numbers were red using an Electrical Conductivity (E.C.) meter model JENWAY4510, and a pH meter model METROHM691. Particle density, bulk density, and total porosity were measured based on Chen et al. [34]. The field capacity and permanent wilting point of the substrate was measured according to the method of Salter and Haworth [35]. Organic carbon and organic matter measurements were based on Walkley and Black [36]. The digestion method determined macro and microelements. An instrument Inductively Coupled Plasma-Optical Emission Spectrometer (ICP-OES) Model 76,004,555 made in Germany, was used to take these measurements (Table 1). Throughout the study period, data on relative humidity, wind speed, precipitation, and air temperature at the experiment site were meticulously recorded (Fig. 4).

**Table 1:** Some physical and chemical properties of the studied substrate

substrate	N (%)	P (%)	K (%)	Mg (%)	Ca (%)	Fe (mg.kg)	Zn (mg.kg)	Cu (mg.kg)	Mn (mg.kg)	Na (mg.lit)	HCO3 (meq.lit)	OC (%)	OM (%)	Ash (%)	FC (%)	PWP (%)	EC (ds/m)	pH
25 cocopeat + 5% vermicompost + 55% perlite + 10% vermiculite + 5% zeolite	0.082	0.05	0.40	0.73	1.4	2.1	0.4	1.1	2.3	45	1.2	4.03	2.34	90	121.77	13.08	1.74	6.95

HCO3 (Bicarbonate ion), OC (Organic carbon), OM (Organic matter), FC (Field Capacity), PWP (Permanent wilting point), EC (Electrical conductivity), pH (acidity)

### Measurements

#### Morphological traits

The measured morphological traits in this experiment included plant height, internode distance (measured using a ruler), stem diameter, leaf diameter, leaf length, leaf width (measured using a digital caliper), number of lateral branches, number of nodes, and number of leaves on lateral branches. These measurements were taken on a monthly basis [37, 38]. The growth index (plant width × plant length × plant height) was also assessed on a monthly basis.

### Plant coverage

The plant surface coverage, also known as the horizontal and vertical coverage of plants, was calculated using quadrat constructed to the size of 20 × 20 square centimeters for each experimental unit in each green wall. Each quadrat consisted of 100 chambers, with each chamber measuring 2 × 2 in width and length.

### Physiological and Biochemical Traits

All physiological and biochemical traits were evaluated at the end of the experiment during the autumn season.

### Total chlorophyll and carotenoid

The total chlorophyll concentration was assessed using the method described by Dere [39]. Fresh leaves weighing 0.2 g were thoroughly ground and homogenized in a mortar with 10 mL of 96% methanol. The grinding and homogenization process should be carried out in a cool and low-light environment. The resulting mixture was filtered through filter paper, and then subjected to centrifugation at 2500 rpm for 10 min. The supernatant was immediately collected, and the light absorption at wavelengths of 666 nm, 653 nm, and 470 nm was measured using a spectrophotometer for chlorophyll a, chlorophyll b, and carotenoids, respectively. Finally, the carotenoid and total chlorophyll concentrations were determined using the Eqs. (3) and (4):

1 $$\mathrm{Chl\,a}\left(\mathrm{\mu g}/\text{ml}\right)=\left(15/\times\mathrm A666\right)-(7/34\times\mathrm A653)$$

2 $$\mathrm{Chl\,b}\left(\mathrm{\mu g}/\mathrm{ml}\right)=\left(27/05\times\mathrm A653\right)-(11/21\times\mathrm A666)$$

3 $$\mathrm{Carotenoid}\left(\mathrm{\mu g}/\mathrm{ml}\right)=\left(1000\times\mathrm A470\right)-\left(2/860\times\mathrm{Chla}\right)-(129/2\times\mathrm{Chlb})/245$$

4 $${\text{CHL}}_{t}= \text{Chl a}+\text{Chl b}$$

### Relative Water Content (RWC)

The calculation of leaf relative water content (RWC) was performed using the method described by Hossain et al., [40]. Initially, a leaf sample was weighed using a balance to obtain the initial weight. Then, to obtain the turgid weight, the samples were placed in closed containers containing distilled water for 12 h at a temperature of 21 °C (19 to 23 °C). After removing excess water from the leaf surface, the turgid weight of the samples was measured. To determine the dry weight, the samples were transferred to an oven at a temperature of 70 °C for 48 h (Eq. 5).

5 $$\text{RWC}= \frac{Fw-Dw}{Tw-Dw}\times 100$$

In which Fw represents the fresh weight, Dw represents the dry weight, and Tw represents the turgid weight of the leaf.

### Relative Saturation Deficit (RSD)

The relative saturation deficit was calculated using the method described by Samar Raza et al., [41]. After collecting the leaves and weighing wet weight them (Fw), they are submerged in distilled water at room temperature for 5 h. Then, the leaves were removed from the water, re-weighed, and their turgid weight (Tw) is obtained. The relative saturation deficit is calculated using Eq. 6.

6 $$\mathrm{RSD}\;\left(\%\right)\;=\left(\mathrm{Tw}-\mathrm{Fw}\right)/\;\mathrm{Tw}\;\times\;100$$

### Electrolyte Leakage (EL)

The stability of the cell membrane was measured using the method described by Sairam and Srivastava [42]. Leaf segments measuring 2 cm in size were prepared and washed. These segments were then placed in test tubes along with 10 ml of distilled water. At this stage, the electrical conductivity of the test samples (E1) was measured using a JENWAY conductivity meter. Then, the test tubes were transferred to an autoclave and subjected to a temperature of 121 degrees Celsius for 15 min to kill the leaf cells. After cooling down, the electrical conductivity was measured again (E2) in this stage. Finally, the electrolyte leakage values were calculated using Eq. 7.

7 $$\text{EL}=\left({\text{E}}_{1}/{\text{E}}_{2}\right) \times 100$$

### Soluble carbohydrates

To measure the soluble carbohydrates, 2.0 ml of methanol extract were mixed with 3 ml of anthrone reagent (0.15 g anthrone in 100 ml of 72% sulfuric acid). The mixture was then placed in a hot water bath at a temperature of 100 degrees Celsius for 20 min to allow the reaction to occur. The absorbance of the samples was measured at a wavelength of 620 nm using a spectrophotometer after cooling [43].

### Total Protein Content

To measure the protein concentration, 5 ml of urine reference solution were added to a test tube, followed by the addition of 100 µl of protein extract, and it was quickly mixed. After 5 min, the absorption was read at a wavelength of 595 nm using a spectrophotometer. The protein concentration was calculated using the standard curve of albumin [44].

### Anthocyanins

To measure anthocyanins in the leaves, the method described by Nadernejad et al., [45] was used. Fresh plant tissue weighing 0.1 g was ground in a mortar and pestle with 10 ml of methanol acid solution (pure methanol and pure hydrochloric acid in a volumetric ratio of 1:99). The extract was poured into a screw-capped test tube and kept in darkness at a temperature of 25 °C for 24 h. Then, it was centrifuged at 4000 rpm for 10 min, and the absorbance of the supernatant was measured at a wavelength of 550 nm using a spectrophotometer. The concentration was calculated using Eq. 8, considering the extinction coeffici of 33,000 (ε) in cm/mol (Eq. 8).

8 $$\mathrm A=\mathrm{\epsilon bc}$$

where A represents absorbance, b is the cell width, and c is the concentration of the solution under investigation.

### Total phenols

Determination of total phenols was performed using the Folin-Ciocalteu reagent at a wavelength of 765 nm, following the method described by Singleton and Rossi [46]. The measurement was carried out by calibrating the standard curve with gallic acid, and the amount of total phenolic compounds was expressed as milligrams of gallic acid equivalents per 100 g of dry weight.

### Malondialdehyde (MDA)

To measure malondialdehyde (MDA) levels, approximately 0.2 g of fresh leaf tissue (the youngest leaves at the tip of the stem) were ground in a mortar containing 5 ml of 0.1% tri-chloroacetic acid (TCA). To the resulting centrifuged solution (1 ml), 5 ml of 20% TCA solution containing 5.0% thiobarbituric acid (TBA) were added. The concentration of malondialdehyde was measured at a wavelength of 532 nm. As other compounds besides malondialdehyde in the solution exhibit non-specific absorption, their absorption at a wavelength of 600 nm was also measured [47] (Eq. 9).

9 $$\text{A}=\text{\pounds bc}$$

In Eq. (9), A represents the absorption of the sample of interest, £ represents the molar absorptivity coefficient, which is equal to 1.55 × 10^–5^ Mcm^−1^, and C represents the concentration of malondialdehyde.

### Assays of antioxidant activities

To measure the antioxidant activity, the methanolic extract was first diluted at a ratio of 1:10. Then, to deactivate the free radicals, 4 ml of 2,2-Diphenyl-1-picrylhydrazyl (DPPH) solution was added to each sample [48]. The samples were kept in darkness for 30 min, and the absorbance of the resulting solutions and the absorbance of the control sample were measured at a wavelength of 517 nm using a spectrophotometer. The inhibition percentage of DPPH was obtained by comparing the absorbance of the extract sample with the absorbance of the control sample using Eq. 10.

10 $$\%\mathrm{AA}:\;\left[\left(\mathrm{Absorbance}\;\mathrm{of}\;\mathrm{the}\;\mathrm{control}\;\mathrm{sample}\;-\;\mathrm{Absorbance}\;\mathrm{of}\;\mathrm{the}\;\mathrm{evaluated}\;\mathrm{sample}\right)/\;\mathrm{Absorbance}\;\mathrm{of}\;\mathrm{the}\;\mathrm{control}\;\mathrm{sample}\right]\times100$$

### Assay GPX activity

To evaluate GPX activity, initially, 2 ml of 0.05 M sodium phosphate buffer with a pH of 5.6 were mixed with 2 ml of 3% hydrogen peroxide and 2 ml of 5-micromolar ascorbate in an ice bath. Immediately, 1.0 ml of enzyme extract from leaf tissue was added, and the absorbance changes at 256 nm were monitored by a spectrophotometer for 2 min with 10 s intervals. The enzyme activity is defined as the volume of enzyme required to hydrolyze one millimole of substrate per minute at 25 °C. Then, the enzyme activity was calculated in units per minute per milligram of protein [49, 50].

### Assay PPO activity

To measure the activity of the PPO enzyme, pyrogallol was used as the enzyme substrate. The reaction mixture consisted of 2.5 ml of 50 mM potassium phosphate buffer (pH 7), 200 µl of 0.02 M pyrogallol, and 100 µl of enzyme extract. The absorbance of the samples was read at a wavelength of 420 nm after three minutes using a spectrophotometer. The enzyme activity was calculated using the molar absorptivity coefficient of 6.2 Mm^−1^Cm^−1^ [51].

### Assay CAT activity

The CAT activity was measured by Kendal and Scandellius [52] method. Initially, 5.2 ml of 0.05 M potassium phosphate buffer with a pH of 7 and 0.3 ml of 3% hydrogen peroxide were mixed together in an ice bath. Immediately, 0.2 ml of enzyme extract were added to the mixture, and the absorbance changes at a wavelength of 240 nm were monitored for 4–3 min. Enzyme unit was defined per H_2_O_2_ µmol ml-^1^ decomposed per minute at 25 °C and then the enzyme activity was calculated in terms of unit changes per minute per mg of protein.

### Assay APX activity

To evaluate APX activity, according to this method, initially, 2 ml of 0.05 M sodium phosphate buffer with a pH of 5.6 were mixed with 2 ml of 3% hydrogen peroxide and 2 ml of 5-micromolar ascorbate in an ice bath. Immediately, 1.0 ml of enzyme extract from leaf tissue was added, and the absorbance changes at 256 nm were monitored by a spectrophotometer for 2 min with 10 s intervals. The enzyme activity is defined as the volume of enzyme required to hydrolyze one millimole of substrate per minute at 25 °C. Then, the enzyme activity was calculated in units per minute per milligram of protein [50].

### Assay POX activity

Measurement of POX activity was done according to Holley's method [53]. In this regard, initially, 2 ml of 0.2 M acetate buffer with a pH of 5, 0.2 ml of 3% hydrogen peroxide, and 0.1 ml of a 0.02 M benzidine solution in 50% methanol are mixed in an ice bath. Then, 0.1 ml of leaf enzyme extract is added to this mixture, and the absorbance curve of the samples is plotted using a spectrophotometer at a wavelength of 530 nm, at room temperature, every 30 s for 3 min. The specific enzyme activity is calculated by using the standard curve and determining the change in enzyme unit per minute per milligram of protein.

### Statistical analysis

JMP 8 software was used for statistical analysis. Data analysis was performed using analysis of variance (ANOVA) and mean comparison with the Tukey test at a significance level of at least 5%. All graphs were plotted using Excel software.

## Results

According to the analysis of variance, the simple effects of recycled water treatments showed significant differences at the 5% probability level in the growth index characteristics of *A. cordifolia*. For plant height, only the simple effects of bacterial strains were statistically significant at the 5% level. When evaluating the number of lateral branches, significant differences were observed in both the interaction effects of the two treatments and the individual effects of bacterial strains (p ≤ 0.05). The simple effects of irrigation water type and bacterial strain showed significant differences at 5% probability in surface coverage and RSD. Additionally, in the case of total chlorophyll, RWC, carotenoids, and EL, not only the simple effects of irrigation and bacteria but also their interaction effects were significant at 5% level (p ≤ 0.05) (Table 2).

**Table 2:** The mean square results from the variance analysis of the effect of different treatments on the type of irrigation water and the type of bacteria on some of the Morphophysiological characteristics of *Aptenia cordifolia*

Sources of Variation	df	Height	Growth index	lateral branch length	number of lateral branches	Surface coverage	Total chlorophyll	Carotenoid	RWC	RSD	EL
Block	2	15.08	2.54e^+8^	20.02	1.36	35,364	0.01	0.64	18.07	8.42	39.26
Irrigation	2	99.75^ns^	1.33e^+9^^b^	581.36^a^	15.02^ ns^	132,132^b^	1.80^a^	12.28^a^	1076.42^a^	781.03^a^	1124.76^a^
Main factor error	4	27.95	1.62e^+8^	9.27	4.27	8288	0.01	0.29	52.17	48.33	37.104
Bacteria	3	571.21^a^	7.48e^+8 ns^	125.28^a^	0.01^b^	115,938^b^	0.56^a^	4.80^a^	396.52^a^	212.30^b^	4015.23^a^
Iirrigation × Bacteria	6	84.71^ ns^	5.20e^+8 ns^	52.50^b^	0.0003^a^	51,640.7^ ns^	0.10^b^	6.03^a^	60.54^b^	46.88^ ns^	232.05^b^
Sub factor error	18	78.51	304,827,392	16.49	3.37	31,950.4	0.04	0.89	19.86	18	75.07

^a^, ^b^: significant at 1% and 5% probability levels, *ns* non-significant, *df* degrees of freedom. Data analysis was used JMP 8 software and analysis of variance (ANOVA) as split plots based on a completely randomized block design. RWC (Relative Water Content), EL (Electrolyte Leakage), RSD (Relative Saturation Deficit)

### Type of irrigation water and *bacteria* on morphophysiological traits of Aptenia cordifolia

#### The impact of varied irrigation water and bacterial strains on the growth traits and surface coverage

According to the analysis of variance, all morphological traits of the plant species were statistically significant at 5% probability level.

### Plant height and growth index

**Fig. 5:**
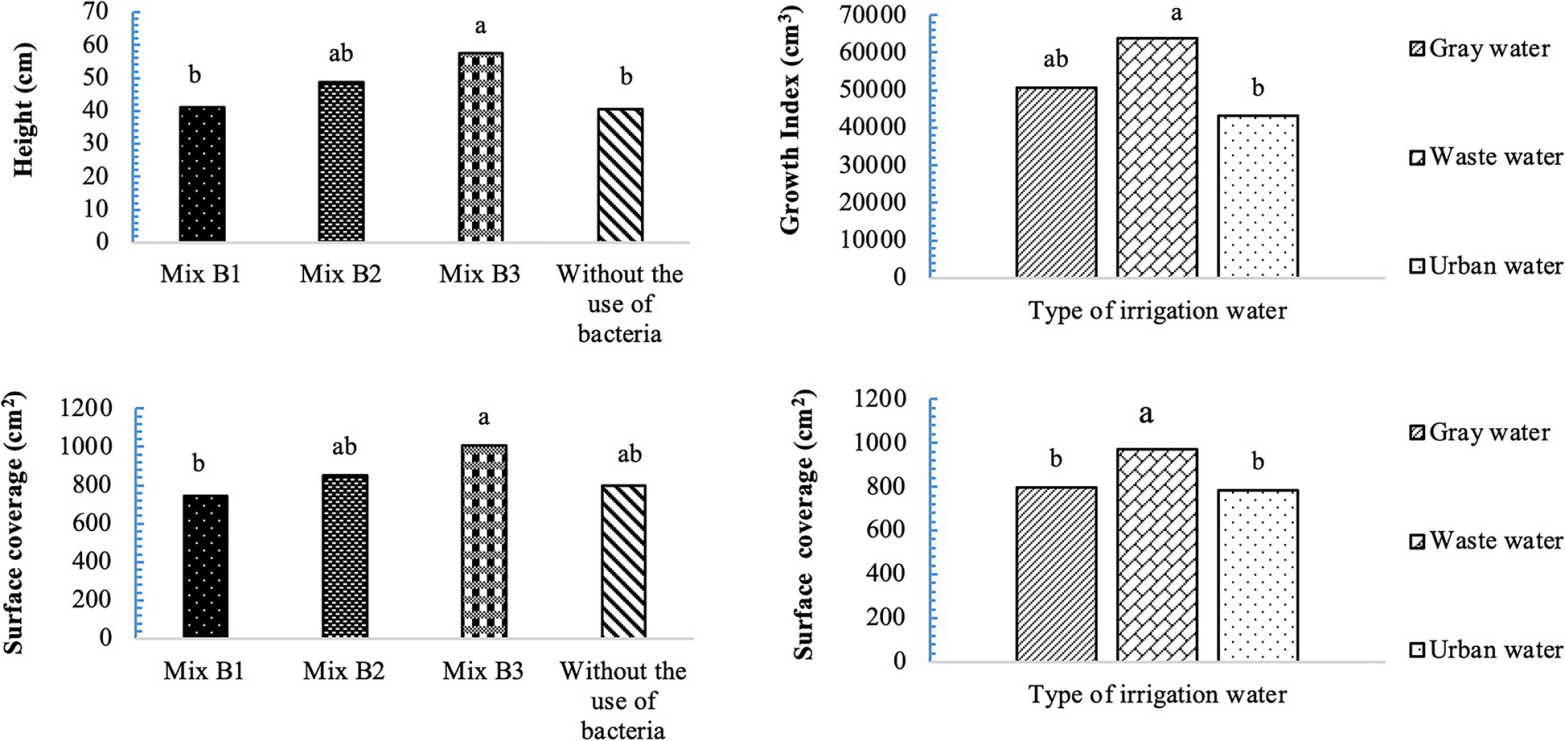
Simple effects of recycled water and bacterial strains on growth traits, Plant height: (standard error: 2.95). Growth index: (standard error: 3575.99) and coverage level, the data are shown A and B (standard error: 1.06, 2.34). Data analysis was used JMP 8 software and analysis of variance (ANOVA) as split plots based on a completely randomized block design

As shown in Fig. 5, the simple effects of bacterial strains on the height of the studied plant species in ice plant indicate that inoculation of the substrates with Mix B3 and Mix B2 resulted in an increase in plant height. Regarding the growth index in the ice plant, irrigation with wastewater and gray water resulted in a higher growth index compared to urban water.

As evident from the analysis of variance (Table 3), in relation to the biochemical data of *A. cordifolia*, the simple effects of bacterial strain treatments showed significant differences at 1% probability level in proline, GPX activity, and CAT. However, the simple effects of irrigation water type and bacterial strain showed significant differences at 1% level in POX activity. In the evaluation of carbohydrates and antioxidant activity, apart from the interactive effect of the two treatments, there were significant differences in the simple effects of bacterial strains (p ≤ 0.05). Additionally, in leaf phenolic, besides the simple effects of irrigation water type (p ≤ 0.01), the block effect and their interaction effects also showed significant differences (p ≤ 0.01). According to the analysis of variance (Table 3), regarding the total protein, anthocyanins, MDA, PPO enzyme, and APX, apart from the simple effects of irrigation water type and bacterial strain (*p* ≤ 0.01), their interaction effects also showed significant differences (p ≤ 0.05).

**Table 3:** The mean square results from the variance analysis of the effect of different treatments on the type of irrigation water and the type of bacteria on some of the Biochemical characteristics of *Aptenia cordifolia*

Sources of Variation	df	carbohydrate	Total protein	Anthocyanin	Total leaf phenol	MDA	Proline	Antioxidant activity	GPX	PPO	CAT	APX	POX
Block	2	81.58	128.59	0.50	5.37^a^	5.97	0.32	56.52	2.03e^−6^	5.84e^−5^	0.17	0.22	0.10
Irrigation	2	372.17^ ns^	1136.7^b^	2.99^b^	4.61*	126.94^b^	0.84^ ns^	94.36^ ns^	5.25e^−6 ns^	0.003^a^	0.10^ ns^	1.65^a^	1.53^a^
Main factor error	4	222.53	75.05	0.21	0.27	8.62	0.16	56.80	1.7e^−6^	0.00001	0.07	0.045	0.05
Bacteria	3	3678.77^a^	1373.58^a^	44.03^a^	2.33^ ns^	305.18^a^	5.06^a^	548.09^a^	0.00008^a^	0.013^a^	0.23a	6.02^a^	1.72^a^
Irrigation × Bacteria	6	427.14^b^	1368.18^a^	5.46^a^	7.97^a^	37.07^a^	0.42^ ns^	204.99^b^	8.21e^−6 ns^	0.002^a^	0.03^ ns^	5.30^a^	0.21^ ns^
Sub factor error	18	112.15	103.44	0.32	1.52	4.48	0.21	52.13	9.72e^−6^	0.0001	0.03	0.09	0.17

^a^, ^b^: significant at 1% and 5% probability levels, *ns* non-significant, *df* degrees of freedom. Data analysis was used JMP 8 software and analysis of variance (ANOVA) as split plots based on a completely randomized block design

### Surface coverage

As observed in Fig. 3, the treatment involving the inoculation of the substrates with Mix B3 and irrigation with wastewater led to increased surface coverage (262.67 cm2) in the ice plant. It is noteworthy that the Mix B1 resulted in less surface coverage compared to the control (without bacterial application), suggesting that the B1 combination was not particularly successful in *A. cordifolia*.

### Impact of varied irrigation water and bacterial strains on the number and length of lateral branches

**Fig. 6:**
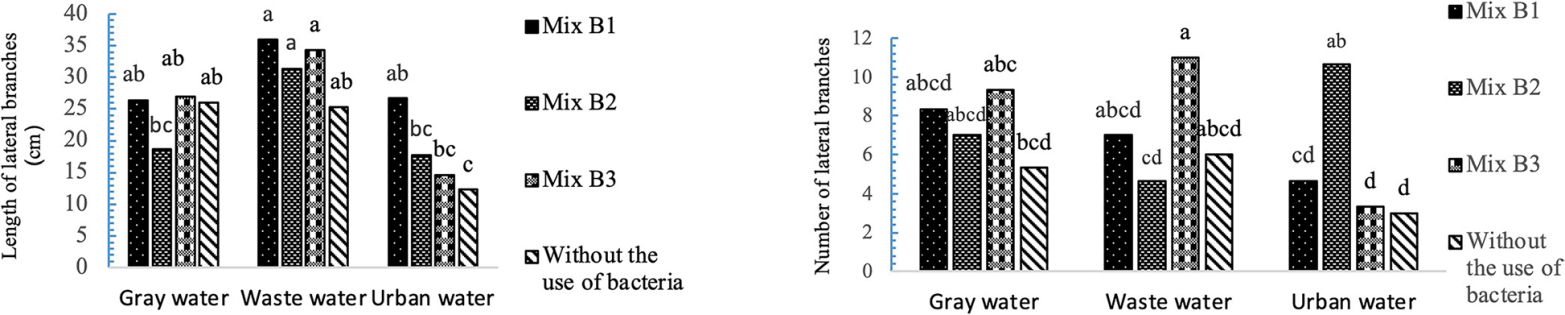
Interaction effects of recycled water and bacterial strains on n the number and length of lateral branches data are shown (standard error: 1.06, 2.34). Data analysis was used JMP 8 software and analysis of variance (ANOVA) as split plots based on a completely randomized block design

Figure 6 demonstrates that the highest number of lateral branches in *A. cordifolia*. (11 branches) was observed in the presence of Mix B3 and irrigation with wastewater. The lowest number of lateral branches in the ice plant was observed in tap water in the presence of Mix B3 and B0, with an average of approximately 3 branches (Fig. 6). Additionally, the maximum lateral branch length in *A. cordifolia*. in all three water type treatments was related to the presence of the combined strains B3, B2, and B1, and the lowest was related to the control.

**Fig. 7:**
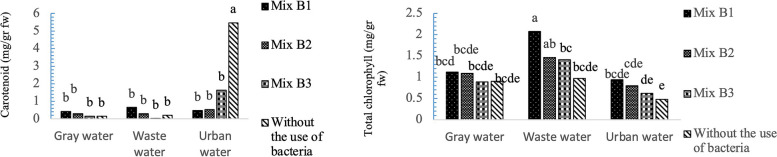
Interaction effects of recycled water and bacterial strains on photosynthetic pigments data are shown total chlorophyll: (standard error: 0.11). Carotenoid: (standard error: 0.54). Data analysis was used JMP 8 software and analysis of variance (ANOVA) as split plots based on a completely randomized block design

According to Fig. 7, the highest total chlorophyll content was observed in Mix B1 in plants irrigated with wastewater (2.07 mg/g, FW), while the lowest total chlorophyll content was associated with the B0 of plants irrigated with urban water (0.62 mg/g, FW). Regarding carotenoid content in the *A. cordifolia*, the control treatment (without bacterial application) using urban water exhibited the highest carotenoid levels. Plants treated with the combined bacterial strains showed less leaf yellowing across various irrigation water types.

### Impact of varied irrigation water and bacterial strains on RWC

**Fig. 8:**
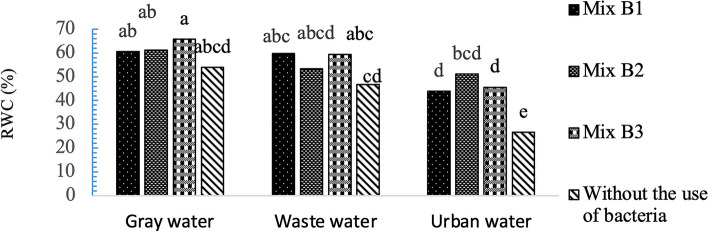
Interaction effects of recycled water and bacterial strains on RWC, (standard error: 2.57). Data analysis was used JMP 8 software and analysis of variance (ANOVA) as split plots based on a completely randomized block design

According to Fig. 8, in *A. cordifolia*, inoculation with Mix B3 in the substrate and irrigation with gray water resulted in the highest leaf water content (65.68%). The lowest leaf water content was observed in the control treatment (without bacterial application) and irrigation with urban water (26.69%).

### Impact of varied irrigation water and bacterial strains on RSD

**Fig. 9:**
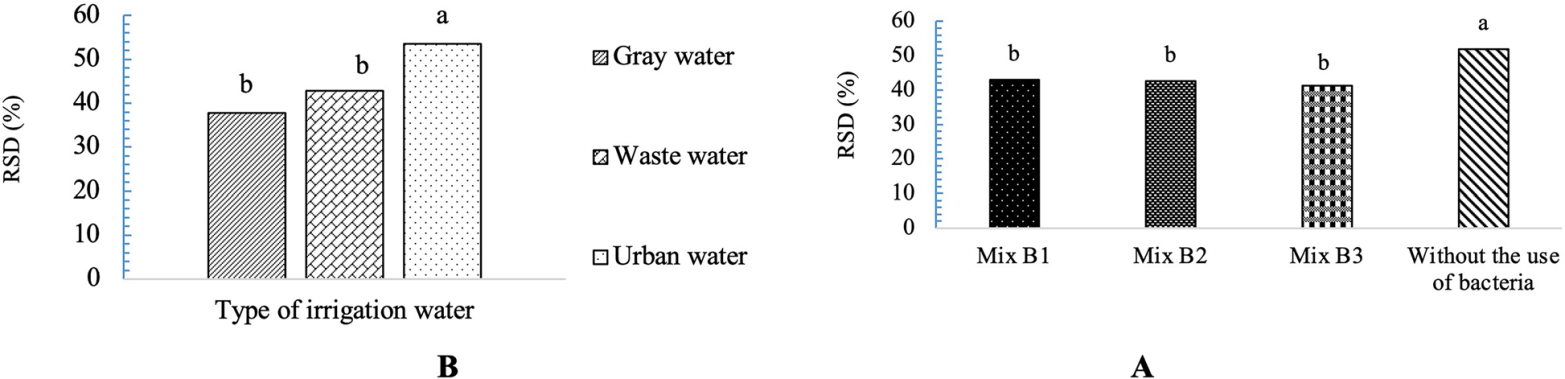
Interaction effects of recycled water and bacterial strains on RSD, data are shown **A** and **B**: (standard error: 1.41, 2). Data analysis was used JMP 8 software and analysis of variance (ANOVA) as split plots based on a completely randomized block design

As evident in Fig. 9, the highest relative water deficit in *A. cordifolia* was associated with the control treatment (without bacterial application). It is noteworthy that the highest RSD in the experiment was also observed in plants irrigated with urban water. It appears that recycled waters resulted in a lower RSD.

### Biochemical characteristics of ornamental plant

Different small Latin letters in each column of the table indicate a significant difference based on Tukey's test at a probability level of at least 5%. Data represent ± 1 standard error (SE). Data analysis was used JMP 8 software and analysis of variance (ANOVA) as split plots based on a completely randomized block design.

GPX: (guaiacol peroxidase), POD: (peroxidase), CAT: (catalase), B1:)*Sedoumonas flucrecens* + *Azosporillum Liposferum* + *Thiobacillus thioparus* + *Aztobactor chorococcum*), B2: (*Paenibacillus polymyxa* + *Pseudomonas fildensis* + *Bacillus subtilis* + *Achromobacter xylosoxidans* + *Bacillus licheniformis*), B3: (*Pseudomonas putida* + *Acidithiobacillus ferrooxidans* + *Bacillus velezensis* + *Bacillus subtilis* + *Bacillus methylotrophicus* + *Mcrobacterium testaceum*) and B0: without the use of bacteria.

### The impact of varied irrigation water and bacterial strains on soluble carbohydrates

According to Table 4, in *A. cordifolia*, the highest soluble carbohydrates content was associated with Mix B3 and irrigation with wastewater (62.82 mg/g FW). On the other hand, urban water in the control treatment (without bacterial application) and Mix B1 in gray water and urban water exhibited similar behavior and resulted in the lowest soluble carbohydrates content (4.18 mg/g FW).

**Table 4:** Comparison of the means of the interaction effects of recycled water and bacterial strains on the biochemical properties of *Aptenia cordifolia*

Type of irrigation water		carbohydrate (mg per gram fresh weight of leaf)	Total protein (mg per gram fresh weight of leaf)	total phenol (mg per gram fresh weight of leaf)	Anthocyanin (micromole per gram wet weight)	EL (%)	MDA (micromole per gram wet weight)	Antioxidant activity (%)	PPO (unit^−1^ mg protein)	APX (unit^−1^ mg protein)
Gray water	B1	4.24d ± 0.28	5.75bc ± 0.003	4.81ab ± 1.05	0.52de ± 0.08	76.72 ab ± 1.92	17.52ab ± 0.72	38.05abc ± 0.14	0.005c ± 0.0004	1.69de ± 0.11
	B2	28.17bcd ± 0.30	18.35bc ± 0.009	5.11a ± 0.17	3.02b ± 0.04	53.91 bc ± 4.81	0.82gh ± 0.33	53.12a ± 1.34	0.013c ± 6.67e^−5^	2.84bc ± 0.17
	B3	57.93ab ± 11.28	14.27bc ± 0.006	1.62ab ± 0.10	7.93a ± 1.71	22.43 de ± 2.20	12.46bcde ± 1.25	38.44abc ± 4.96	0.023c ± 0.0002	0.94ef ± 0.32
	B0	15.96 cd ± 0.70	0.16c ± 0.001	2.37ab ± 0.66	0.59de ± 0.81	83.50 a ± 2.83	16.49ab ± 0.49	36.32abc ± 1.23	0.0097c ± 0.0144	2.83bc ± 0.16
Wastewater	B1	26.28bcd ± 4.21	18.90bc ± 0.001	1.52ab ± 0.21	0.78de ± 0.07	77.20 ab ± 3.01	8.46def ± 0.54	27.85bc ± 0.43	0.0072c ± 9.64e^−5^	2.35 cd ± 0.15
	B2	37.86abc ± 1.57	25.38bc ± 0.001	2.12ab ± 1.38	2.12 cd ± 0.43	53.21 bc ± 3.55	7.10efg ± 0.09	36.24abc ± 0.91	0.017c ± 0.0002	2.10 cd ± 0.13
	B3	62.82a ± 9.35	81.70a ± 0.015	4.24ab ± 0.54	4.66b ± 1.20	35.19 cde ± 3.03	0.45 h ± 1.98	54.20a ± 9.78	0.14a ± 0.0002	4.21a ± 0.12
	B0	8.42 cd ± 1.18	10.20bc ± 0.002	1.68ab ± 0.19	1.56de ± 0.48	83.56 a ± 4.18	18.90a ± 0.49	27.27bc ± 1.89	0.010c ± 0.0001	0.38f ± 0.04
Urban water	B1	5.02d ± 1.09	27.98bc ± 0.002	1.44ab ± 0.06	0.82de ± 0.06	48.04 cd ± 5.12	15.19abc ± 0.80	34.76abc ± 2.11	0.0091c ± 7.04e^−5^	0.84ef ± 0.12
	B2	57.93ab ± 6.34	14.27bc ± 0.006	1.62ab ± 0.27	1.44de ± 0.50	55.81bc ± 7.50	12.46bcde ± 0.12	38.44abc ± 1.79	0.023c ± 0.0002	0.94ef ± 0.05
	B3	39.82abc ± 7.89	13.72bc ± 0.020	2.69ab ± 0.72	3.45bc ± 0.43	21.33 e ± 1.14	2.68fgh ± 2.19	37.64abc ± 0.18	0.087b ± 0.0744	3.61ab ± 0.06
	B0	4.18d ± 0.75	12.05bc ± 0.000	1.33b ± 0.13	0.23e ± 0.23	55.23 bc ± 2.42	13.77abcd ± 1.33	22.19c ± 2.55	0.0058c ± 2.17e^−5^	0.28f ± 0.12

*MDA*: (malondialdehyde), *PPO* (polyphenol oxidase), *APX* (ascorbate peroxidase), B1(Sedoumonas flucrecens + Azosporillum Liposferum + Thiobacillus thioparus + Aztobactor chorococcum), B2: (Paenibacillus polymyxa + Pseudomonas fildensis + Bacillus subtilis + Achromobacter xylosoxidans + Bacillus licheniformis), B3: (Pseudomonas putida + Acidithiobacillus ferrooxidans + Bacillus velezensis + Bacillus subtilis + Bacillus methylotrophicus + Mcrobacterium testaceum) and B0: without the use of bacteria

### The impact of varied irrigation water and bacterial strains on total protein

In *A. cordifolia*, the highest total protein content was observed the substrate inoculated with Mix B3 and plants irrigated with wastewater (81.70 mg/g FW). The lowest total protein content was found in the control treatment (without bacterial application) and irrigation with gray water (0.16 mg/g FW). It appears that gray water was not very successful in enhancing cellular sap thickening in *A. cordifolia.*

### The impact of varied irrigation water and bacterial strains on total phenol

The highest total phenolic content was observed in the substrate inoculated with Mix B2 and plants irrigated with wastewater in *A. cordifolia* (5.11 mg/g FW). However, the lowest content was found in the control treatment (without bacterial application) and irrigation with tap water (1.33 mg/g FW) (Table 4).

### The impact of varied irrigation water and bacterial strains on anthocyanin

In *A. cordifolia*, the highest anthocyanin was observed in the substrate inoculated with Mix B3 and plants irrigated with gray water (7.93 μmol/g FW), while the lowest content was found in the control treatment (without bacterial application) and irrigation with urban water (0.52 μmol/g FW). It appears that the inoculation of the substrate with Mix B3 leads to an increase in anthocyanins in Plants of the CAM family.

### The impact of varied irrigation water and bacterial strains on MDA

The MDA content was significantly affected by the main effects and interaction effects of different irrigation water types and bacterial strains (Table 3). The application of wastewater led to an increase in the MDA content in plants under irrigation (18.90 μmol/g FW), indicating a degree of tissue damage in the plant. In most cases, the MDA content decreased with bacterial treatments. The highest reduction in MDA content was observed, in order, with Mix B3, Mix B2, and irrigation with wastewater (0.82 μmol/g FW, 0.45 μmol/g FW).

### The impact of varied irrigation water and bacterial strains on proline

In *A. cordifolia*, the control treatment (without bacterial application) and Mix B1 resulted in higher proline levels. The presence of Mix B3 and Mix B2 performed better in reducing proline. According to Table 5, statistically, the three types of water sources had a similar effect and did not show any specific statistical differences. It is important to note that Mix B1 acted similarly to the control treatment in reducing proline stress indicators. It appears that Mix B1 may not be effective in reducing the stress caused by unconventional water sources in *A. cordifolia* (Table 5).

**Table 5:** Comparison of the means of the simple effects of recycled water and bacterial strains on the the biochemical properties of *Aptenia cordifolia*

Treatments	Proline	GPX (unit^−1^ mg protein)	CAT (unit^−1^ mg protein)	POD (unit^−1^ mg protein)
Gray water	1.75a ± 0.19	0.008a ± 0.0011	0.35a ± 0.051	1.53a ± 0.13
Wastewater	1.75a ± 0.26	0.009a ± 0.0009	0.53a ± 0.071	0.92b ± 0.15
Urban water	1.75a ± 0.25	0.009a ± 0.0010	0.43a ± 0.089	0.91b ± 0.15
B1	1.98a ± 0.16	0.008ab ± 0.0010	0.47ab ± 0.093	0.81b ± 0.06
B2	1.13b ± 0.28	0.012a ± 0.0005	0.34b ± 0.056	1.07b ± 0.16
B3	0.56b ± 0.14	0.010a ± 0.0008	0.29b ± 0.040	1.75a ± 0.15
B0	2.17a ± 0.18	0.005b ± 0.0009	0.65a ± 0.093	0.85b ± 0.20

Different small Latin letters in each column of the table indicate a significant difference based on Tukey's test at a probability level of at least 5%. Data represent ± 1 standard error (SE). Data analysis was used JMP 8 software and analysis of variance (ANOVA) as split plots based on a completely randomized block design

### The impact of varied irrigation water and bacterial strains on antioxidant activity

The results obtained from ANOVA indicated that different bacterial strains had a significant effect (at a minimum level of 5%) on the activity of GPX, PPO, CAT, APX, and POX enzymes (Table 3). The application of different bacterial strains increased the activity of GPX, PPO, CAT, APX, and POX enzymes (Tables 4 and 5). Table 4 showed that in the ice plant, the highest antioxidant activity was observed in Mix B2 and Mix B3 and irrigation with wastewater (53.12% and 54.20%, respectively). In contrast, irrigation with municipal wastewater and tap water in the control treatment (without bacteria) exhibited the lowest antioxidant activity (22.19%).

Bacterial inoculation, in most cases, increased the activity of the four antioxidant enzymes in different irrigation water types (Tables 4 and 5). The highest increase in PPO and APX activity was observed, respectively, with Mix B3 and irrigation with wastewater (0.14 unit^−1^ mg protein) and (4.21 unit^−1^ mg protein). The lowest level of PPO activity was observed in the control treatment (without bacteria) and Mix B2 and Mix B1 in all three water types. Regarding APX, the lowest activity was associated with the control treatment (without bacteria) and irrigation with wastewater and urban water (Table 4).

Comparing the mean data shows that the substrate inoculated with Mix B2 and Mix B3 was associated with the highest GPX activity (0.012 and 0.01 unit^−1^ mg protein, respectively), while the lowest activity of this enzyme was observed in Mix B1 and the control medium (B0) (0.005 unit^−1^ mg protein). CAT activity exhibited an opposite trend compared to the other enzymes in this study, where Mix B1 and the control medium (B0) had the highest CAT activity (0.65 unit^−1^ mg protein) (Table 5). Additionally, the highest increase in POX activity was observed with Mix B3 (1.75 unit^−1^ mg protein), while other bacterial combinations did not significantly improve POX activity and were similar to the control. On the other hand, irrigation of plants with graywater resulted in increased POX activity (1.53 unit^−1^ mg protein) (Table 5).

## Discussion

Water scarcity has necessitated the use of unconventional water sources for agricultural. The issue of water crisis is a significant concern globally, particularly in Middle Eastern countries, where climate change and global warming are exacerbating the problem [54]. Overcoming this scarcity, farmers resort to irrigating their crops with treated or untreated wastewater due to its low cost and availability.

### The impact of varied irrigation water and bacterial strains on morphological factors of *Aptenia* cordifolia in external green wall

The persistent application of recycled water has the potential to deteriorate soil quality and contribute to the accumulation of toxic heavy metals in the food chain [55]. This decline in soil quality caused by unconventional water sources results in reduced quality and quantity of crops grown in these soils and can be considered a form of stress for plants [56]. However, as observed in the present experiment, the presence of the mentioned microbial strains in wastewater not only did not reduce the quality and quantity of the cultivated plants compared to tap water but also improved certain traits (height, growth index, number and length of lateral branches, chlorophyll, leaf content, RWC, RSD and EL). These results are consistent with the findings of Urbano et al. (2017) [57] regarding increased plant growth due to higher nutrient content compared to freshwater, which can serve as a nutrient source for agriculture and reduce the demand for chemical fertilizers. Furthermore, studies have shown that plant growth-promoting bacteria enhance productivity in various environmental conditions, especially under stressful situations, to improve the detrimental effects of unconventional water on the quantitative and qualitative traits of plants [58]. In the present study, it was also demonstrated that the application of bacteria in the substrate resulted in improvement of the adverse effects of these unconventional waters, although the performance of all bacterial treatments was not identical. Mix B3 took the top position, followed by Mix B2 in the subsequent ranking. These bacteria appear to stimulate plant growth through nutrient provision, secretion of plant growth hormones, and various other mechanisms associated with PGPR [59]. In this study, an increase in chlorophyll content in all tested plants resulted in higher photosynthetic productivity, ultimately leading to better growth, increased plant organs, and improved surface coverage by the plant. The extent of surface coverage depends on the growth characteristics of plants, such as height, length, and width, which are influenced by nutrient conditions and substrate moisture [60]. Jozay et al., [61] also stated that the composition and content of substrate significantly affect the growth and coverage of plants on external green surfaces. Rapid coverage in vertical green structures, such as green walls, is a desirable trait. The utilization of bacterial combinations has proven effective in enhancing this characteristic. Kumar et al., [62] reported that the combined use of sewage sludge and plant growth-promoting rhizobacteria resulted in improved performance (*Luffa acutangula* (L.) Roxb), with the highest fresh biomass (9.6 ± 0.3 g), growth rate (1.4 ± 0.1 g/day), plant length (15.5 ± 0.3 cm), root length (10.4 ± 0.3 cm) and total chlorophyll (3.2 ± 0.1 mg/g). The findings of this study regarding the increase in growth indices of the studied plants in the presence PGPB and irrigation align with the results of the mentioned researchers.

It can be said that *A. cordifolia* (Mesembryanthemum crystallinum) has been studied in favorable conditions in all four seasons and due to its greenness, trailing habit, and sufficient coverage to create a desirable visual landscape, it can be used for its visual attractiveness and appeal in combination substrates used in exterior green walls. Kazemi et al., [63] examined the growth and performance of four plant species in various substrate types in indoor green wall systems, and the results indicated that *A. cordifolia* is a desirable species for indoor green wall systems. One of the influential factors in the aesthetic performance of green wall systems is the percentage of plant coverage, which is dependent on plant growth indices, and this finding aligns with the study by Kazemi et al., [63] regarding the application of *A. cordifolia* and the current research results.

The leaf chlorophyll content has a close relationship with leaf nitrogen content in plants and can serve as an indicator of nitrogen availability in the soil [64]. Lubbe et al., [65] highlighted a decrease in chlorophyll content in the leaves of *Amarantus dubius* and *Solanum nigrum* under greywater irrigation treatments, suggesting that greywater irrigation may jeopardize nutrient accessibility compared to tap water irrigation. Vajpayee et al., [66] assessed the chl a/b ratio among plants exposed to different concentrations of tannery effluents and reported a greater reduction in chlorophyll-a compared to chlorophyll-b. The maximum reduction in carotenoid content in *Spirodela polyrhiz*a was observed after 7 days at a concentration of 75% tannery effluent. The present results support the degradation of carotenoids due to increased effluent in the soil and metal toxicity. In this study, an increase in pigments was observed in wastewater and greywater in the presence of PGPB, a trend that contradicted the significant reduction in photosynthetic pigments observed with effluent application. In present research, greywater and effluent irrigation resulted in increased plant greenness, which may be attributed to the presence of PGPB.

Yadav and Pandey [67] also observed a positive correlation between leaf relative water content and the concentration of chlorophyll, protein, and ribisco activity. Leaf water content in plants helps maintain physiological water balance under unfavorable environmental conditions. Jozay et al., [68] stated that there is a direct relationship between leaf water content and resistance to environmental stress conditions in PGPR (Plant Growth-Promoting Rhizobacteria). Plants inoculated with bacterial strains have the ability to modify lateral root system architecture and increase RWC [69]. Increased relative water content (RWC) in stressed leaves of plants inoculated with plant growth-promoting rhizobacteria (PGPR) has also been reported in other studies [70]. In general, an increase in the number of lateral roots and root hairs adds surface area for nutrient and water uptake. Enhanced water and nutrient uptake by inoculated roots improve the water status of plants, which in turn can be a primary factor in promoting plant growth [71].

The RSD impacts the water relations of plants, including the water content of plant tissues and gas exchange in leaves, leading to an increase in the relative water content (RWC) of leaves and a decrease in transpiration and leaf stomatal conductance [72]. Optimal growth conditions, along with efficient nutrient absorption and transport to plants, enhance the accumulation of ions and organic molecules in leaf vacuoles. This contributes to maintaining the water balance by reducing leaf osmotic potential [73]. Vishnupradeep et al., [74] found that MST-PGPB inoculation increased RWC under various stress conditions. PGPB can maintain water potential to prevent water loss by reducing root surface drying. Likewise, Woo et al., [75] suggested that PGPB inoculation improves RWC through various PGP metabolites, including siderophore production, ACC deaminase activity, phosphate solubilization, and IAA synthesis, resulting in enhanced plant tolerance to abiotic stress, biomass production, and protein content, particularly under non-biological stress conditions [76]. Substrate inoculated with Mix B3 and Mix B2, leading to an increase in pigments and RWC, as well as a reduction in RSD, confirming the findings of the mentioned researchers.

### Impact of varied irrigation water and bacterial strains on Biochemical factors of *Aptenia* cordifolia in external green wall

Previous studies have reported an increase in growth associated with PGPB and an increase in secondary metabolites and anthocyanins [77]. The microbial population has a positive correlation with plant biomass, antioxidant enzyme activity, and anthocyanins, while it has a negative correlation with the production of free radicals [78], indicating the contribution of rhizosphere microbial population in reducing oxidative stress through increased anthocyanin production and enhanced antioxidant enzyme activity. In plants, the greatest decrease in proline and MDA content and the highest increase in antioxidant enzyme activity were observed substrate inoculated with Mix B3 and Mix B2. Singh and Malaviya [79] reported that due to acute toxicity of chromium present in wastewater, anthocyanins in the effluent were below detectable levels after 4 days. The increase in anthocyanins in this study, despite repeated use of wastewater and graywater, could be attributed to the presence of bacterial strains. The results of this study, which showed an increase in anthocyanins through substrate inoculated with Mix B3 in irrigated vegetable plants with recycled water, are consistent with the reports of Pagnani et al., [80] and Kumar et al., [62].

The result indicates that the increase in proline was in response to watering the plants with municipal wastewater. Proline may act as an osmolyte under stress conditions and increase proline activities thereby minimizing the side effects of stress [81]. In other findings, Islam et al., [82] reported that proline acts as a growth regulator and also protects cells against ROS accumulation. It is thought that proline is able to reduce the negative effects of cadmium toxicity on plant growth in plant tissues and reduce oxidative stress [83]. In this study, the use of growth-promoting bacteria reduced the adverse effects of wastewater irrigation, and it is worth noting Mix B3 and Mix B2 was more successful in reducing the amount of proline than the other two treatments. As a result, it can be said the use of growth-promoting bacteria has put the plant in a favorable condition by increasing the growth index and biomass and has reduced stress and proline content.

Proline and free phenols are non-enzymatic antioxidants that support organisms in unfavorable conditions by reducing the undesirable effects of reactive oxygen species (ROS) [84]. Environmental stresses, such as heavy metal exposure in unconventional water sources and urban gardening, have been documented to increase ROS production. Antioxidant molecules and enzymes play a crucial role in detoxifying ROS in plant cells. Antioxidant compounds, including proline and phenols, inhibit oxidation and play vital roles in stress responses [85]. After irrigating plants with wastewater in the control treatment without bacteria, the levels of these compounds significantly increased, confirming the findings of the aforementioned researchers [86]. It has been reported that the content of phenolic compounds tends to rise when plants are subjected to heavy metal stress, as phenolic compounds act as scavengers of reactive oxygen species and metal chelators. In contrast, the content of free proline and total phenols considerably decreased in response to our treatments compared to plants irrigated with water in the presence of growth-promoting bacteria. The highest reduction was observed in free proline when substrate inoculated with Mix B3 and Mix B2. These results indicate the role of these treatments in reducing the toxic effects of wastewater on plants. Our findings align with previous studies by Liu et al., [12], which reported that wastewater treatment with bacteria significantly reduces the stress caused by metal contaminants in wastewater. Regarding total phenols, the control treatment in urban water showed the highest reduction. The decrease in phenolic content may be attributed to oxidative stress, while a further increase in the content of phenolic compounds (as non-enzymatic antioxidants) was observed when substrate inoculated with Mix B3 and wastewater. These results are consistent with the reports of Khodamoradi et al., [87] regarding the reduction of phenols under stress conditions.

Malondialdehyde (MDA) is a byproduct of lipid oxidation and is responsible for cellular membrane damage, inducing alterations in membrane radical properties. These changes ultimately culminate in cell death [88]. In this study, the MDA content increased in response to wastewater irrigation in the control treatment B0. This may be attributed to the oxidative system's inability to reduce ROS levels, thereby failing to prevent damage to the cell membrane. This result is also supported by Yildirim et al., [89], who demonstrated that irrigation with contaminated water generally increased the MDA in plants. In contrast, the MDA content significantly decreased in response to treatments B3 and B2 compared to plants irrigated with wastewater in treatment B0. This may be due to the reduction in ROS production and the enhancement of the antioxidant system and repair mechanisms. These results are supported by a recent study by Malik et al., [90].

Some studies have found that PGPB strains can enhance plant growth and mitigate the negative effects of various stresses on plant growth. Enhanced growth associated with PGPB and increased secondary metabolites, carbohydrates, total protein, and antioxidant potential have been reported in Astragalus mongholicus [91]. Plant antioxidant enzymes GPX, PPO, CAT, APX, POX act as the first line of defense for tolerating unfavorable conditions. These enzymes stimulate the detoxification of ROS and reduce the detrimental effects of non-biological stress [91]. Another finding is that in most cases, bacterial treatments increased the activity of antioxidant enzymes. The greatest increase in biomass production and antioxidant enzyme activity was observed in substrate inoculated with Mix B3 and Mix B2 in wastewater, indicating a significant contribution of bacterial inoculation in activating antioxidant enzymes and promoting plant growth. The further increase in GPX, PPO, APX, and POX activities resulting from the most effective bacterial inoculation may indicate the major role of these enzymes in improving plant biomass through substrate inoculated with Mix B3.

Under wastewater stress, plants exposed to a stronger antioxidant system are less exposed to free radicals, leading to lower MDA production. High antioxidant activity in stress-tolerant plants may indicate their ability to neutralize harmful oxidants and maintain their growth and productivity at a normal level. Therefore, it can be concluded that these bacteria, in addition to promoting growth, provide new perspectives for the development of biofertilizers to alleviate environmental stresses. It can be inferred that greater protection was achieved under wastewater irrigation when bacterial strains were applied. Ultimately, Mix B3 and Mix B2 appear to be recommendable to farmers due to their economic and environmental compatibility, aiding in mitigating the adverse effects of recycled water use in urban gardening.

Phytoremediation is an environmentally friendly and cost-effective alternative to removing pollution from soil. Due to the non-degradable chemical nature of heavy metals in soil, we need to understand the function of antipollution facilitators such as plant growth promoting bacteria to improve or facilitate the removal of heavy metals by plants [92, 93]. Heavy metal tolerant PGPR have also increased plant growth in heavy metal contaminated soil in recent years. These PGPR have successfully played an important role in promoting plant growth while reducing toxicity or damage to plants exposed to stress produced by various heavy metals in soil [94]. PGPR produce plant growth regulators, phytohormones, and various secondary metabolites that promote plant growth and reduce heavy metal toxicity. Various mechanisms are employed by PGPR to enhance plant growth under heavy metal stress. Many of them lead to the reduction of heavy metal toxicity [95].

Known mechanisms by which PGPR can benefit plants under a variety of stresses include: (1) bioremediation of heavy metal-contaminated soils by sequestering toxic heavy metal species and improving soil structure (by bacterial exopolysaccharides); (2) the synthesized enzyme ACC (1aminocyclopropane-1-carboxylate) deaminase, an enzyme that is involved in the reduction of stress-induced ethylene levels in the roots of growing plants; (3) supply of N2 to the plant through biological nitrogen fixation. (4) Production of siderophores. (5) production of phytohormones (such as ABA (abscisic acid), GA (gibberellic acid), auxin, for example, indole-3-acetic acid (IAA) and CK (cytokinin); (6) control of plant pathogens by various mechanisms such as the production of extracellular enzymes that hydrolyze the fungal cell wall, competition for nutrients in the rhizosphere, induction of systemic resistance (ISR), and production of antibiotics and siderophores; (7) dissolution and mineralization of nutrients, especially inorganic phosphate; and (8) improving resistance to abiotic stresses [92, 96].

Therefore, growth-promoting bacteria, as living organisms, play an important role in plant nutrition balance. They can help improve their nutrition by reducing environmental stress and increasing the digestion and absorption of nutrients in plants. Using growth-promoting bacteria as an alternative to chemical fertilizers for soil repair and plant nutrition can be an effective method. These bacteria are commonly known as biologicals or biofertilizers. The use of microorganisms can reduce the stresses in various plants in stressed soils, thus opening a potential and promising strategy for sustainable agriculture [97].

In a recent report by Jozay [92], it was suggested that PGPR could be used as a bioremediation method for soils contaminated with toxic metals. PGPR contain bacteria that are rhizospheric and endophytic and facilitate bioremediation. Plants accumulate heavy metals in the roots and reduce their transfer to other parts of the plant. These microorganisms provide benefits to plants by providing nutrients and reducing the harmful effects of pollutants.

As pointed out by FAO [98], promoting safe and healthy agricultural practices in urban environments is essential to achieve sustainable urban development. At the same time, environmental pollutants in cities should be controlled while using city resources and inputs. The purpose of this study is to investigate the potential of green wall systems to produce horticultural materials that are aligned with the goals of sustainable urban development.

## Conclusion

The results of this study indicated that plant irrigation using wastewater significantly increased the content of free proline, total phenol, GPX, PPO, CAT, APX, POX and MDA compared to the control. On the other hand, the PGPB reduced the oxidative damage effects of wastewater irrigation by increasing antioxidant activity and enhancing GPX, PPO, APX, POX. As a result, the content of free proline, total phenol, and MDA decreased significantly, while carbohydrates, proteins, and anthocyanins increased in the combined bacterial treatments. It can be concluded that better protection under wastewater irrigation was achieved using our treatments. Ultimately, Mix B3 and Mix B2 economically and environmentally friendly, can be recommended to farmers for mitigating the adverse effects of reclaimed water on urban gardening. Substrate inoculated with Mix B3, wastewater irrigation, and subsequent greywater irrigation, affects water availability, nutrient availability, and physiological traits of ornamental plants, including RWC, RSD, and plant pigment properties, leading to freshness and increased greening of the plant. It also has a considerable effect on morphological traits such as height, growth index, lateral branches, and surface coverage, and there are significant differences in plant growth improvement among the combined substrate inoculated with different bacteria types. This treatment can be used to improve the qualitative and morphological traits of ornamental plants used in external green walls under similar climatic conditions to Mashhad city. Water scarcity is expected to transform the reuse of reclaimed water for irrigation into a widespread and common practice globally. The incorporation of growth-promoting bacteria can serve as aneffective amendment for mitigating the toxicity associated with wastewater, facilitating improved plant growth in areas irrigated with wastewater and greywater. As a sustainable solution, green walls have the potential to mitigate water, soil, and air pollution, thereby enhancing environmental sustainability. By incorporating technology into multifunctional green walls, we can take a significant step towards sustainable urban development. Living green walls are not only introduced as effective tools for urban space management but also as instruments for enhancing the climate resilience of cities. The outcome of this research is the registration of a water recycling system utilizing nature-based methods, with Patent number 110287.

### Future Prospects

Future research should focus on understanding the molecular mechanisms underlying these beneficial interactions and exploring the application of PGPB in other plant species and environmental conditions. Additionally, long-term field studies are needed to evaluate the sustainability and economic viability of using PGPB in urban green wall systems.

## Data Availability

The data that support the findings of this study are available from the corresponding author upon ‎reasonable request.‎

## Abbreviations

B0-3: Strain bacteria 0–3
B0: Without the use of bacteria
B1: *Psedoumonas flucrecens* + *Azosporillum liposferum* + *Thiobacillus thioparus* + *Aztobactor chorococcum*
B2: *Paenibacillus polymyxa* + *Pseudomonas fildensis* + *Bacillus subtilis* + *Achromobacter xylosoxidans* + *Bacillus licheniform*
B3: *Pseudomonas putida* + *Acidithiobacillus ferrooxidans* + *Bacillus velezensis* + *Bacillus subtilis* + *Bacillus methylotrophicus* + *Mcrobacterium testaceum*
OC: Organic carbon
OM: Organic matter
FC: Field capacity
PWP: Permanent wilting poin

## References

[1] V´asquez A, Giannotti E, Gald´amez E, Vel´asquez P, Devoto, C. Green Infrastructure planning to tackle climate change in Latin American cities. In C. Henriquez, & H. Romero (Eds.), Urban Climate in Latin-American Cities. 2019;32–354. Springer.

[2] Langemeyer J, Connolly JJT. Weaving notion of justice into urban ecosystem service research and practice. Environ Sci Policy. 2020;109:1–14. 10.1016/j.envsci.2020.03.021

[3] Artmann M, Sartison K. The role of urban agriculture as a nature-based solution: A review for developing a systemic assessment framework. Sustainability. 2018;10:1937. 10.3390/su10061937

[4] Cojocariu M, Chelariu EL, Chiruţă C. Study on Behavior of Some Perennial Flowering Species Used in Vertical Systems for Green Facades in Eastern European Climate. Appl Sci. 2022;12(1):474. 10.3390/app12010474

[5] Mahmoudi Jabri K, Nolde E, Ciroth A, Bousselmi L. Life cycle assessment of a decentralized greywater treatment alternative for non-potable reuse application. Int J Environ Sci Tech. 2020;17(1):433–44. 10.1007/s13762-019-02511-3

[6] Jozay M, Rabbani Khairkhah M, Kazemi F. The impact of humic acid solutions and types of growing media on some morphophysiological and biochemical features of Syngonium sp and Pothos sp plants in interior green wall conditions. Plant Arshive. 2021;21:2240–52.

[7] Savi T, Casolo V, Luglio J, Bertuzzi S, Trifilo P, Lo Gullo M.A, ardini, A. Species-specific reversal of stem xylem embolism after a prolonged drought correlates to endpoint concentration of soluble sugars. Plant Physiology and Biochemistry. 2016; 106:198–207. 10.1016/j.plaphy.2016.04.051 27174138

[8] Jozay M. Rabbani Khairkhah SM. Vertical green systems (green wall). Iran Publishing House (Aristo Publications). 2019.

[9] Li X, Chang Z, Lian X, Meng G, Ma J, Guo R, Wang Y. Phytoremediation of cadmium contaminated alkaline soil using the ornamental hyperaccumulator Mirabilis jalapa L. enhanced by double harvesting: a field study. Environmental Science and Pollution Research. 2022; 1–8. 10.1007/s11356-022-18589-1 35029826

[10] Chouhan S, Agrawal L, Prakash A. Amelioration in traditional farming system by exploring the different plant growth-promoting attributes of endophytes for sustainable agriculture. Arch Microbiol. 2022;204:151. 35075529 10.1007/s00203-021-02637-4

[11] Singh P, Singh RK, Zhou Y, Wang J, Jiang Y, Shen N, Wang Y, Yang L, Jiang M. Unlocking the strength of plant growth promoting Pseudomonas in improving crop productivity in normal and challenging environments: a review. Journal of Plant Interactions. 2022;17(1):220–38. 10.1080/17429145.2022.2029963

[12] Liu Sh, Liu H, Chen R, Ma Y, Yang B, Chen Zh, Liang Y, Fang J, Xiao Y. Role of Two Plant Growth-Promoting Bacteria in Remediating Cadmium-Contaminated Soil Combined with Miscanthus floridulus (Lab). Plants. 2021;10:912. 34063227 10.3390/plants10050912 PMC8147505

[13] Xiao Y, Liu H, Chen R, Liu S, Hao X, Fang J. Heteroauxin-producing bacteria enhance the plant growth and lead uptake of Miscanthus floridulus (Lab.). International Journal of Phytoremediation. 2022; 1–8. 10.1080/15226514.2021.2024134 34995152

[14] Gavrilescu M. Enhancing phytoremediation of soils polluted with heavy metals. Curr Opin Biotechnol. 2022;74:21–31. 34781102 10.1016/j.copbio.2021.10.024

[15] Zanin G, Bortolini L, Borin M. Assessing stormwater nutrient and heavy metal plant uptake in an experimental bioretention pond. Land. 2018;7:150. 10.3390/land7040150

[16] Zhuang J, Qiao L, Zhang X, Su Y, Xia Y. Effects of visual attributes of flower borders in urban vegetation landscapes on aesthetic preference and emotional perception, Int. J. Environ. Res. Publ. Health. 2021; 18(17):9318. 10.3390/ijerph18179318, 3. 10.3390/ijerph18179318 PMC8431206 34501927

[17] Brix H, Arias CA. The use of vertical flow constructed wetlands for on-site treatment of domestic wastewater: new Danish guidelines. Ecol Eng. 2005;25:491–500. 10.1016/j.scitotenv.2015;09.154. 10.1016/j.scitotenv.2015;09.154

[18] Silvestri G, Aliata F. El paisaje como cifra de armonía, Ediciones Nueva Visi´on, Buenos Aires. 2001.

[19] Noutsopoulos C, Andreadakis A, Kouris N, Charchousi D, Mendrinou P, Galani A, Mantziaras I, Koumaki E. Greywater characterization and loadings, physicochemical treatment to promote onsite reuse. J Environ Manage. 2018;216:337–46. 28592390 10.1016/j.jenvman.2017.05.094

[20] Soudi B. Appui a La Promotion De La Reutilisation Des Eaux Usees Par Le Renforcement Des Aspects Institutionnels, Reglementaires Et Financieres, Ainsi Que Des Démarches Participatives, Des Mesures Incitatives Et La Sensibilisation (Activité n_EFS-MO-2). 2018. Available online: https://www.swim-h2020.eu/wp-content/uploads/2018/09/SWIM-H2020-EFS-MO-2.

[21] Rosa PAL, Galindo FS, Oliveira CE dS, Jalal A, Mortinho ES, Fernandes GC, Marega EMR, Buzetti S, Teixeira Filho MCM. Inoculation with Plant Growth-Promoting Bacteria to Reduce Phosphate Fertilization Requirement and Enhance Technological Quality and Yield of Sugarcane. Microorganisms. 2022;10:192. 10.3390/microorganisms10010192 PMC8781176 35056643

[22] National Centers for Climatology. Climate information of the city of Mashhad, 2019. available on: https://www.ncdc.noaa.gov/climate-information, access date: 2019.07.25.

[23] F.L.L. Guidelines for the planning, construction and maintenance of green roofing, Forschungsgesellschaft Landschaftsentwic. Bonnklung Landschaftsbau e.V.-FLL, Bonn. 2018.

[24] Khorshid M, Oustan S, Najafi N, Khataee A. Kinetic characterization of hexavalent chromium stabilization in contaminated soils amended with cocopeat. Arab J Geosci. 2020;13(11):1–12. 10.1007/s12517-020-05421-8

[25] Wang F, Miao L, Wang Y, Zhang M, Zhang H, Ding Y, Zhu W. Using cow dung and mineral vermireactors to produce vermicompost for use as a soil amendment to slow Pb2+ migration. Appl Soil Ecol. 2022;170: 104299. 10.1016/j.apsoil.2021.104299

[26] Gong Y, Zhang G, Hao Y, Nie L. Enrichment Evaluation of Heavy Metals from Stormwater Runoff to Soil and Shrubs in Bioretention Facilities. Water. 2022;14(4):638. 10.3390/w14040638

[27] Bai G, Luo F, Zou Y, Liu Y, Wang R, Yang H, Liu Z, Chang J, Wu Z, Zhang Y. Effects of vermiculite on the growth process of submerged macrophyte Vallisneria spiralis and sediment microecological environment. J Environ Sci. 2022;118:130–9. 10.1016/j.jes.2021.08.038 35305761

[28] Yu G, Wang G, Chi T, Du C, Wang J, Li P, Zhang Y, Wang S, Yang K, Long Y, Chen H. Enhanced removal of heavy metals and metalloids by constructed wetlands: A review of approaches and mechanisms. Sci Total Environ. 2022;821:153516. 35101517 10.1016/j.scitotenv.2022.153516

[29] Ekhlasi Nia A. Evaluation of arsenic and iron transport from sediments of a potable water treatment plant wastewater stabilization pond system. M.Sc. Thesis, In the Department of Civil, Geological and Environmental Engineering, Canada. 2021.

[30] Huang Q, Qiu W, Yu M, Li S, Lu Z, Zhu Y, Kan X, Zhuo R. Genome-Wide Characterization of Sedum plumbizincicola HMA Gene Family Provides Functional Implications in Cadmium Response. Plants. 2022;11(2):215. 35050103 10.3390/plants11020215 PMC8779779

[31] Ju W, Liu L, Fang L, Cui Y, Duan Ch, Wu H. Impact of co-inoculation with plant-growth-promoting rhizobacteria and rhizobium on the biochemical responses of alfalfa-soil system in copper contaminated soil. Ecotoxi & Environl Safety. 2019;167:218–26. 10.1016/j.ecoenv.2018.10.016 30342354

[32] Esfandiari M, Hakimzadeh Ardakani MA, Sodaiezadeh H. Investigation of heavy metal Contamination in the ornamental plants of green spaces in the city of Yazd. Journal of Ornamental Plants. 2020;10(4):213–22.

[33] Allison LE, Moodie CD. Carbonate. p. 1379–1400. In C.A. Black et al. (Ed.) Methods of Soil Analysis. Part 2. 2nd Ed. Agron. Monogr. 9. ASA, CSSA, and Soil Science Society of America, Madison, WI. 1965.

[34] Chen Y, Inbar Y, Hadar Y. Composted agricultural wastes as potting media for ornamental plants. Soil Sci. 1988;145(4):298–303. 10.1097/00010694-198804000-00009

[35] Salter PJ, Haworth F. The available water capacity of a sandy loam soil: I. A critical comparison of methods of determining the moisture content of soil at field capacity and at the permanent wilting percentage. Journal of Soil Science. 1961;12:326–34. 10.1111/j.1365-2389.1961.tb00922.x

[36] Walkley A, Black TA. An examination of Deglijareff method for determining soil organic matter and a proposed modification of the choromic acid titration method. Soil Sci. 1934;37:29–38. 10.1097/00010694-193401000-00003

[37] Mozaffari S, Khorasaninejad S, Gorgini SH. The effects of irrigation regimes and humic acid on some of physiological and biochemical traits of Common Purslane in greenhouse. Journal of Crops Improvement (Journal of Agriculture). 2017;19(2):401–16.

[38] Gorgini Shabankareh H, Khorasaninejad S, Sadeghi M, Tabasi AR. The effects of irrigation periods and humic acid on morpho- physiological and biochemical traits of Thyme (*Thymus vulgaris*). Journal of Plant Ecophysiological Research. 2018;13(51):67–82.

[39] Dere S, Günes T, Sivaci R. Spectrophotometric determination of chlorophyll-a, b and total carotenoid contents of some algae species using different solvents. Turk J Bot. 1998;22:7–13.

[40] Hossain MI, Khatun A, Talukder MSA, Dewan MMR, Uddin MS. Effect of drought on physiology and yield contributing characters of sunflower. Bangladesh J Agric Res. 2010;35(1):113–24. 10.3329/bjar.v35i1.5872

[41] Samar Raza MA, Saleem MF, Khan IH, Jamil M, Ijaz M, Khan MA. Evaluating the drought stress tolerance efficiency of wheat (Triticum aestivum L) Cultivars. Russ J Agri Socio-Econ Sci. 2012;12(12):41–6.

[42] Sairam RK, Srivastava GC. Changes in antioxidant activity in sub-cellular fractions of tolerant and susceptible wheat genotypes in response to long term salt stress. Plant Sci. 2002;162:897–904. 10.1016/S0168-9452(02)00037-7

[43] Sadasivam S, Manickam A. In: Biochemical Methods for Agricultural Sciences. New Delhi: Wiley Eastern Ltd.; 1992. p. 184–5.

[44] Bradford MM. A rapid and sensitive method for the quantitation of microgram quantities of protein utilizing the principle of protein-dye binding. Anal Biochem. 1976;72(1–2):248–54. 942051 10.1016/0003-2697(76)90527-3

[45] Nadernejad N, Ahmadimoghadam A, Hossyinifard J, Poorseyedi S. Study of the rootstock and cultivar effect in PAL activity, production of phenolic and flavonoid compounds on flower, leaf and fruit in Pistachio (*Pistacia vera* L). Journal of Plant Biology. 2013;15:95–110.

[46] Singleton VL, Rossi JA. Colorimetry of total phenolics with phosphomolybdicphosphotungstic acid reagents. Am J Enol Vitic. 1965;16:144–58. 10.5344/ajev.1965.16.3.144

[47] Heath RL, Packer L. Photoperoxidation in isolated chloroplasts: I. Kinetics and stoichiometry of fatty acid peroxidation. Arch Biochem Biophys. 1968;125(1):189–98. 5655425 10.1016/0003-9861(68)90654-1

[48] Moon JH, Terao J. Antioxidant activity of caffeic acid and dihydrocaffeic acid in lard and human lowdensity lipoprotein. J Agric Food Chem. 1998;46:5062–5. 10.1021/jf9805799

[49] Jia L, Xu W, Li W, Ye N, Liu R, Shi L, Bin Rahman AR, Fan M, Zhang J. Class III peroxidases are activated in proanthocyanidin deficient Arabidopsis thaliana seeds. Ann Bot. 2013;111:839–47. 23448691 10.1093/aob/mct045 PMC3631330

[50] Asada K, Takahashi M. Production and scavenging of active oxygen in chloroplasts, In: D.J. Kyle, B. Osmond & C.J. Arntzen (Eds), photoinhibition, Elsevier Amsterdam. 1987;227–287.

[51] Jebara S, Jebara M, Limam F, Aouani ME. Changes in ascorbate peroxidase, catalase, guaiacol peroxidase and superoxide dismutase activities in common bean (Phaseolus vulgaris) nodules under salt stress. J Plant Physiol. 2005;162(8):929–36. 16146319 10.1016/j.jplph.2004.10.005

[52] Chandlee JM, Scandalios JG. Analysis of variants affecting the catalase developmental program in maize scutellum. Theor Appl Genet. 1984;69:71–7. 24253626 10.1007/BF00262543

[53] Holy MC. Indole acetic acid oxidase: a dual catalytic enzyme. Plant Phisiology. 1972;50:15–8. 10.1104/pp.50.1.15 PMC367307 16658111

[54] Rezapour S, Kouhinezhad P, Samadi A. The potential ecological risk of soil trace metals following over five decades of agronomical practices in a semi-arid environment. Chem Ecol. 2017;34:68–78.

[55] Zemanová V, Trakal L, Ochecova P, Száková J, Pavlíková D. A model experiment: competitive sorption of Cd, Cu, Pb and Zn by three different soils. Soil and Water Research. 2014;9:97–103. 10.17221/50/2013-SWR

[56] Kanwal A, Farhan M, Sharif F, Hayyat MU, Shahzad L, Ghafoor GZ. Effect of industrial wastewater on wheat germination, growth, yield, nutrients and bioaccumulation of lead. Sci Rep. 2020; 10:11352–11361. 10.1038/s41598-020-68208-7. 10.1038/s41598-020-68208-7 PMC7347546 32647263

[57] Urbano VR, Mendonça TG, Bastos RG, Souza CF. Effects of treated wastewater irrigation on soil properties and lettuce yield. Agric Water Manag. 2017;181:108–15. 10.1016/j.agwat.2016.12.001

[58] Ramakrishna W, Yadav R, Li K. Plant growth promoting bacteria in agriculture: two sides of a coin. Appl Soil Ecol. 2019;138:10–8. 10.1016/j.apsoil.2019.02.019

[59] Chiappero J, Cappellari Ld. R Sosa Alderete LG, Palermo TB, Banchio E. Plant growth promoting rhizobacteria improve the antioxidant status in Mentha piperita grown under drought stress leading to an enhancement of plant growth and total phenolic content. Industrial Crops and Products. 2019;139:111553.

[60] Rabbani Khairkhah SM, Kazemi F. The effect of the type of cultivation substrate and humic acid on some morphophysiological traits and water consumption of scorpion tail sedum (*Carpobrotus edulis* L) on a green roof system. Journal of Plant Environmental Physiology. 2022;17(66):1–23.

[61] Jozay M, Kazemi F, Fotovat A. The performance of cover plant (*Frankenia thymifolia*) and its effect on the physicochemical properties of the recycled substrates in four different seasons in external green wall systems. Journal of Plant Environmental Physiology. Ready to print publishing. 2022.

[62] Kumar V, Eid EM, Al-Bakre DA, Abdallah SM, Širić I, Andabaka Ž, Kumar P, Goala M, Adelodun B, Singh J, Kumari S. Combined use of sewage sludge and plant growth-promoting rhizobia improves germination, biochemical response and yield of ridge gourd (*Luffa acutangula* (L) Roxb) under field conditions. Agriculture. 2022;12(2):173. 10.3390/agriculture12020173

[63] Kazemi F, Rabbani M, Jozay M. Investigating plant and air-quality performances of an internal greenwall system under hydroponic conditions. Environmental Management. 2020;275:111230. 10.1016/j.jenvman.2020.111230 32861001

[64] Marenco R, Antezana-vera S, Nascimento H. Relationship between specific leaf area, leaf thickness, leaf water content and SPAD-502 readings in six Amazonian tree spe¬cies. Photosynthetica. 2009;47:184–90. 10.1007/s11099-009-0031-6

[65] Lubbe E, Rodda N, Naidoo S. Effects of greywater irrigation on germination, growth and photosynthetic characteristics in selected African leafy vegetables. Water SA. 2016;42(2):203–12. 10.4314/wsa.v42i2.04

[66] Vajpayee P, Sharma SC, Tripathi RC, Rai UN, Yunus M. Bioaccumulation of chromium and toxicity to photosynthetic pigments, nitrate reductase activity and protein content of Nelumbo nucifera Gaertin. Chemosphere. 1999;39:2159–69. 10.1016/S0045-6535(99)00095-8

[67] Yadav R, Pandey P. Assessment of air pollution tolerance index (APTI) and anticipated performance index (API) of roadside plants for the development of greenbelt in urban area of bathinda city, punjab, India. Bull Environ Contam Toxicol. 2020;105:906–14. 33070250 10.1007/s00128-020-03027-0

[68] Jozay M, Kazemi F, Fotovat A. Evaluating the environmental performance of the growing media in a green wall system in a dry climate region. Desert. 2019;20:217–30.

[69] Bertrand A, Gatzke C, Bipfubusa M, Levesque V, Chalifour FP, Claessens A, Rocher S, Tremblay GF, Beauchamp CJ. Physiological and biochemical responses to salt stress of alfalfa populations selected for salinity tolerance and grown in symbiosis with salt-tolerant rhizobium. Agronomy. 2020;10(4):569. 10.3390/agronomy10040569

[70] Zilaie MN, Arani AM, Etesami H, Dinarvand M, Dolati A. Halotolerant plant growth-promoting rhizobacteria-mediated alleviation of salinity and dust stress and improvement of forage yield in the desert halophyte seidlitzia rosmarinus. Environ Exp Bot. 2022;201: 104952. 10.1016/j.envexpbot.2022.104952

[71] Chieb M, Gachomo EW. The role of plant growth promoting rhizobacteria in plant drought stress responses. BMC Plant Biol. 2023;23(1):407. 37626328 10.1186/s12870-023-04403-8 PMC10464363

[72] Anjum SA, Xie XY, Wang L, Saleem MF, Man C, Lei W. Morphological, physiological and biochemical responses of plants to drought stress. Afr J Agric Res. 2011;6:2026–32.

[73] Aeron A, Khare E, Jha CK, Meena VS, Aziz SMA, Islam MT, Kim K, Meena SK, Pattanayak A, Rajashekara H, Dubey RC. Revisiting the plant growth-promoting rhizobacteria: Lessons from the past and objectives for the future. Arch Microbiol. 2020;202(4):665–76. 31781809 10.1007/s00203-019-01779-w

[74] Vishnupradeep R, Bruno LB, Taj Z, Karthik C, Challabathula D, Kumar A, Freitas H, Rajkumar M. Plant growth promoting bacteria improve growth and phytostabilization potential of Zea mays under chromium and drought stress by altering photosynthetic and antioxidant responses. Environ Technol Innov. 2022;25: 102154. 10.1016/j.eti.2021.102154

[75] Woo OG, Kim H, Kim JS, Keum HL, Lee KC, Sul WJ, Lee JH. Bacillus subtilis strain GOT9 confers enhanced tolerance to drought and salt stresses in Arabidopsis thaliana and Brassica campestris. Plant Physiol Biochem. 2020;148:359–67. 32018064 10.1016/j.plaphy.2020.01.032

[76] Rajkumar M, Bruno LB, Banu R. Alleviation of environmental stress in plants: The role of beneficial Pseudomonas spp. Crit Rev Environ Sci Technol. 2017;47:372–407. 10.1080/10643389.2017.1318619

[77] Lin Y, Zhang H, Li P, Jin J, Li Z. The bacterial consortia promote plant growth and secondary metabolite accumulation in Astragalus mongholicus under drought stress. BMC Plant Biol. 2022;22(1):1–14. 36203134 10.1186/s12870-022-03859-4 PMC9541091

[78] Mohsin SM, Hasanuzzaman M, Parvin K, Fujita M. Pretreatment of wheat (Triticum aestivum L) seedlings with 2,4-D improves tolerance to salinity-induced oxidative stress and methylglyoxal toxicity by modulating ion homeostasis, antioxidant defenses, and glyoxalase systems. Plant Physiol Biochem. 2020;152:221–31. 32438299 10.1016/j.plaphy.2020.04.035

[79] Singh A, Malaviya P. Chromium phytoaccumulation and its impact on growth and photosynthetic pigments of Spirodela polyrrhiza (L) Schleid on exposure to tannery effluent. Environmental Sustainability. 2019;2:157–66. 10.1007/s42398-019-00062-4

[80] Pagnani G, Pellegrini M, Galieni A, D’Egidio S, Matteucci F, Ricci A, Stagnari F, Sergi M, Lo Sterzo C, Pisante M, Del Gallo M. Plant growth-promoting rhizobacteria (PGPR) in Cannabis sativa ‘Finola’ cultivation: an alternative fertilization strategy to improve plant growth and quality characteristics. Ind Crops Prod. 2018;123:75–83. 10.1016/j.indcrop.2018.06.033

[81] Khamsuk O, Sonjaroon W, Suwanwong S, Jutamanee K, Suksamrarn A. Effects of 24-epibrassinolide and the synthetic brassinosteroid mimic on chili pepper under drought. Acta Physiol Plant. 2018;40:1–12. 10.1007/s11738-018-2682-z

[82] Islam MM, Hoque MDA, Okuma E, Banu MNA, Shimoishi Y, Nakamura Y, Murata Y. Exogenous proline and glycine betaine increase antioxidant enzyme activities and confer tolerance to cadmium stress in cultured tobacco cells. J Plant Physiol. 2009;166:1587–97. 19423184 10.1016/j.jplph.2009.04.002

[83] Radic S, Babic M, Skobic D, Roje V, Pevalek-Kozlina B. Ecotoxicological effects of aluminum and zinc on growth and antioxidants in Lemna minor L. Ecotoxicol Environ Saf. 2010;73:336–42. 19914715 10.1016/j.ecoenv.2009.10.014

[84] Zandi P, Schnug E. Reactive oxygen species, antioxidant responses and implications from a microbial modulation perspective. Biology (basel). 2022;11:1–30. 10.3390/biology11020155. 10.3390/biology11020155 PMC8869449 35205022

[85] Racchi M. 2013) Antioxidant defenses in plants with attention to prunus and citrus spp. Antioxidants 2:340–369.10.3390/antiox2040340. 10.3390/antiox2040340 PMC4665512 26784469

[86] Pratyusha S. Phenolic compounds in the plant development and defense: an overview. In: Plant stress physiology-perspectives in agriculture. Plant Stress Physiology-Perspectives in Agriculture. 2022;125–185.

[87] Khodamoradi S, Sagharyan M, Samari E, Sharifi M. Changes in phenolic compounds production as a defensive mechanism against hydrogen sulfide pollution in Scrophularia striata. Plant Physiol Biochem. 2022; 177:23–31.10.1016/j.plaphy.2022.02.013. 10.1016/j.plaphy.2022.02.013 35231684

[88] Sharma P, Jha AB, Dubey RS, Pessarakli M. Reactive oxygen species, oxidative damage, and antioxidative defense mechanism in plants under stressful conditions. J Bot. 2012;1–26. 10.1155/2012/217037.

[89] Yildirim E, Ekinci M, Turan M, Agar G, Ors S, Dursun A, Kul R, Balcl T. Impact of cadmium and lead heavy metal stress on plant growth and physiology of rocket (Eruca sativa L). J Agric Nat. 2019; 22:843–850. 10.18016/ksutarimdoga.vi.548626.

[90] Malik Z, Afzal S, Dawood M, Abbasi GH, Khan MI, Kamran M, Zhran M, Hayat MT, Aslam MN, Rafay M. Exogenous melatonin mitigates chromium toxicity in maize seedlings by modulating antioxidant system and suppresses chromium uptake and oxidative stress. Environ Geochem Health. 2022; 44:1451–1469. 10.1007/s10653-021-00908-z. 10.1007/s10653-021-00908-z 33797671

[91] Rai GK, Mushtaq M, Bhat BA, Kumar RR, Singh M, Rai PK. Reactive oxygen species: friend or foe. In: Thermotolerance in crop plants. Springer Nature Singapore, Singapore. 2022;129–162.

[92] Jozay M. Combining Green Space and Urban Gardening in External Green Wall under the Influence of Growth-Promoting Bacteria and Types of Recycled Water. Gorgan, Iran: Gorgan University of Agricultural Sciences and Natural Resources; 2024.

[93] Jozay M, Zarei H, Khorasaninejad S, Miri T. Maximising CO2 Sequestration in the City: The Role of Green Walls in Sustainable Urban Development. Pollutants. 2024;4(1):91–116. 10.3390/pollutants4010007

[94] Etesami H. Bacterial mediated alleviation of heavy metal stress and decreased accumulation of metals in plant tissues: mechanisms and future prospects. Ecotoxicol Environ Saf. 2018;147:175–91. 28843189 10.1016/j.ecoenv.2017.08.032

[95] Etesami H, Maheshwari DK. Use of plant growth promoting rhizobacteria (PGPR) with multiple plant growth promoting traits in stress agriculture: Action mechanisms and future prospects. Ecotoxicol Environ Saf. 2018;156:225–46. 29554608 10.1016/j.ecoenv.2018.03.013

[96] Glick BR. Bacteria with ACC deaminase can promote plant growth and help to feed the world. Microbiol Res. 2014;169:30–9. 24095256 10.1016/j.micres.2013.09.009

[97] Jozay M, Zarei H, Khorasaninejad S, Miri T. The environmental function of external green elements in facade architecture. 8th.International Conference on Researches in Science & Engineering & 5th.International Congress on Civil, Architecture and Urbanism in Asia 24 August. 2023, Kasem Bundit University, Bangkok, Thailand.

[98] FAO. New Standards to Curb the Global Spread of Plant Pests and Diseases; UN Food and Agriculture Organization: Rome, Italy. 2019.

